# Immigrant Wealth Stratification and Return Migration: The Case of Mexican Immigrants in the United States During the Twentieth Century

**DOI:** 10.1215/00703370-10693686

**Published:** 2023-06-01

**Authors:** Mara Getz Sheftel

**Affiliations:** Population Research Institute, Pennsylvania State University, State College, PA, USA;

**Keywords:** Stratification, Wealth, Immigration, Return migration, Life course

## Abstract

Considerable wealth stratification exists between U.S.-born and foreign-born populations (Campbell and Kaufman 2006), with low wealth attainment documented among Mexican immigrants (Hao 2007). High rates of Mexican return migration (Azose and Raftery 2019) suggest that nonrandom selection into return migration on wealth is a potential driver of stratification. Existing theories do not conclusively predict asset accumulation among returnees versus stayers, and empirical research on return migration and wealth stratification is scarce. Combining data from the 2000 U.S. Health and Retirement Study and the 2001 Mexican Health and Aging Study to create a novel data set representing all Mexicans aged 50 and older with a history of migration to the United States and adopting a life course perspective, I find that return migration at younger and older ages is associated with higher wealth accumulation and might be a way to maximize assets at older ages. Thus, return migration may contribute to nativity-based wealth stratification in the United States. The study’s findings point to the greater financial risks for new cohorts of immigrants aging in place, suggest caution in interpreting wealth stratification as a measure of mobility, and inform theories about the links between return migration and wealth across the life course.

## Introduction

Research on wealth stratification by race, ethnicity, and nativity has found low wealth attainment among Mexican immigrants in the United States relative to U.S.-born populations and immigrants from Europe and Asia (Cobb-Clark and Hildebrand 2006; Hao 2007). This wealth gap among Mexican immigrants, the largest foreign-born group in the United States, is concerning. As a critical measure of intergenerational economic security and socioeconomic mobility, wealth has far-reaching implications for ethnoracial stratification into the future. Additionally, because wealth is an important measure of immigrants’ economic integration (Agius Vallejo and Keister 2020), low asset accumulation is considered an indicator of limited integration.

The current understanding of factors contributing to Mexican immigrant wealth stratification does not consider the role of voluntary return migration (Akresh 2011; Campbell and Kaufman 2006; Flippen 2020; Hao 2004; Rugh 2015; Salgado and Ortiz 2020; Valdez 2020), even though Mexican immigrants to the United States represent the largest return migration transit flow globally (Azose and Raftery 2019). In each five-year period between 1990 and 2000, between 670,000 and 870,000 Mexican immigrants to the United States returned to Mexico, a number that grew to more than 1 million between 2010 and 2015 (Azose and Raftery 2019; Gonzalez-Barrera 2015). Thus, research that includes only Mexican immigrants remaining in the United States in analyses of wealth ignores return migration as a potential driver of wealth stratification and results in an incomplete understanding of the life course trajectory of immigrant asset accumulation. Overlooking such processes is inconsistent with classic and contemporary migration theories that offer competing predictions about how wealth attainment and return migration might manifest over the life course (Entwisle et al. 2020).

This article investigates wealth accumulation by return status among Mexican-born individuals with a history of migration to the United States during the twentieth century. Theoretical frameworks point to opposing expectations regarding the economic selection mechanism of voluntary return, complicating hypothesis building. Voluntary return migration can be considered the result of failed economic integration (Todaro 1969). Conversely, voluntary return migration might be an indication of successfully meeting target earning goals to reinvest in the origin country (Stark 1991). Additionally, selection mechanisms may differ by life stage. A life course perspective points to unique characteristics among older immigrants that may differentiate return migration at older versus younger ages, especially in terms of economic outcomes (Durand 2006). In expanding the narrow success–failure framework often applied to selective return migration (de Haas et al. 2015), this article uses a life course perspective and highlights the importance of heterogeneity by life stage in the selection mechanism of return migration (Percival 2013).

To address these gaps in the literature, I use an innovative binational study design,^1^ combining data from two nationally representative data sets: one from the United States and one from Mexico. This approach enables a comparison of asset accumulation at older ages for three Mexican groups with a history of migration to the United States in the twentieth century: *stayers* (Mexican immigrants who stay in the United States into older ages), *younger returnees* (Mexican immigrants to the United States who return to Mexico before age 50), and *older returnees* (Mexican immigrants to the United States who return at age 50 or later). Dividing returnees by age at return also acknowledges different economic reintegration processes that may impact wealth accumulation by older adulthood.

I investigate the relationship between *voluntary* return migration and older adult asset accumulation, noting that the return–asset relationship likely differs for voluntary returnees relative to deportees. Data limitations preclude a direct exclusion of deportees from the sample. However, because 79% of the returnee sample returned before 1986, when deportations sharply increased, I assume that most returnees in the sample did so voluntarily.^2^

I estimate how assets differ between groups using purchasing power parities (PPP) to convert pesos to dollars to account for U.S.-Mexico differences in the cost of living. I find that both returnee groups have a wealth advantage at older ages relative to stayers when adjusting for compositional differences. For younger returnees, this advantage is evident in differences in socioeconomic factors and migration history. For older returnees, the wealth advantage widens after I account for the full range of explanatory variables, suggesting that other processes are at work.

This research makes both empirical and theoretical contributions. To my knowledge, this is the only article providing binational estimates of Mexican immigrant wealth, including both stayers and returnees. Such estimates advance our understanding of stratification processes by clarifying that nonrandom selection into return migration downwardly biases wealth estimates that include only stayers. These estimates also contribute to migration theory in two ways. First, modeling differences in wealth attainment by age at return strengthens our understanding that return migration at older ages is distinct from that at younger ages. Second, this analysis contributes to the theoretical discussion of economic selection mechanisms of return migration, showing that irrespective of the life stage at return, returnees cannot be considered economically failed immigrants. This article lays the descriptive groundwork for future research examining determinants of returnee assets using a causal framework.

## Background

### Immigration and Wealth Stratification

Wealth, defined as net assets, is a comprehensive measure of economic integration and stratification because it incorporates income, consumption, and human capital over the life course (Akresh 2011). Considerable wealth stratification exists between U.S.-born and foreign-born populations (Campbell and Kaufman 2006). Cobb-Clark and Hildebrand (2006) found that the median wealth of U.S.-born couples is 2.5 times that of foreign-born couples. Immigrant Latinos have particularly low asset accumulation relative to other foreign-born groups, and assets of Mexican-born immigrants are among the lowest (Cobb-Clark and Hildebrand 2006; Hao 2007). Foreign-born Mexican individuals report considerably less wealth from intergenerational transfers than U.S.-born Whites, undoubtedly contributing to wealth disparities (McKernan et al. 2014; Salgado and Ortiz 2020).

Evidence suggests that the typical life cycle pattern of wealth accumulation differs for foreign-born versus U.S.-born populations and may partially explain wealth stratification by nativity. Lifetime wealth usually follows an inverted U-shape: wealth accumulates during working ages until retirement, when it is depleted for consumption (Modigliani 1986). For immigrants, time in the United States contributes to life course wealth accumulation because tenure since migration is associated with increased U.S.-specific social and human capital and increased access to labor and financial markets, which may make up for discounted educational attainment in the origin country (Akresh 2011; Hao 2004). Moreover, duration in the United States is associated with place-of-origin economies: immigrants from more economically developed communities stay in the United States longer, motivated by higher returns on their capital than those from economically stagnant areas with more limited opportunities for capital investment (Lindstrom 1996).

Studies have explored several other explanations for lower Mexican immigrant wealth accumulation. First, foreign-born Mexican individuals have lower unadjusted homeownership rates than non-Latino White Americans and third-generation Mexican Americans (Keister et al. 2015). Discriminatory and racist practices in U.S. financial institutions, including predatory lending and higher loan denial rates, hinder immigrants’ access to the real estate market and financial institutions generally (Rugh 2015). Darker-skinned immigrants are particularly vulnerable to these discriminatory practices (Painter et al. 2016). Second, structural barriers, such as lack of access to financial institutions and employment marginality, constrain immigrants’ capital borrowing capacity and impede their wealth accumulation, especially for undocumented immigrants (Flippen 2020). For example, Rugh (2020) found that foreclosure rates are particularly high among undocumented immigrants in Florida, pointing to an erasure of equity even for those who could surmount barriers to homeownership. Third, because small business ownership is a common means of wealth accumulation for immigrants, they may be more vulnerable to changing macroeconomic conditions. Such was the case for Mexican-origin small business owners during the Great Recession (Valdez 2020).

Most data on immigrant wealth do not consider wealth held abroad (Akresh 2011). This omission may be another factor contributing to estimates of low wealth for Mexican immigrants. For example, in a sample of Latino immigrants residing in the United States, Flippen (2020) found that holding assets abroad was almost as common as holding assets in the United States. Although Flippen (2020) and Akresh (2011) included the wealth held abroad of immigrants residing in the United States, to my knowledge, no research has directly considered another transnational process that may contribute to Mexican immigrant wealth stratification: selective return migration.

### Voluntary Return Migration and Economic Outcomes

Theories on return migration and economic outcomes do not conclusively predict asset accumulation among voluntary returnees versus stayers. Neoclassical migration theory focuses on labor supply and demand differences across borders, where wage differences push individuals to migrate in search of higher earnings (Todaro 1969). Underlying this theory are the assumptions that migration is an individual rational decision aiming to maximize income to stay permanently in the host country (Massey et al. 1999) and that return migration represents the failure to integrate into the labor market of the host society (Cassarino 2004; de Haas et al. 2015). This framework points to negative selection of return migrants on economic outcomes, predicting less overall asset accumulation for returnees relative to those remaining in the destination country. Some empirical research on Mexican immigrants in the United States supports this framework, finding that returnees are less successful in terms of wages, skill, and human capital than those remaining in the United States (Chiswick 1980; Lindstrom and Massey 1994; Massey et al. 1990). Such work has not considered wealth, despite its theoretical centrality and its distinctiveness from other indicators.

Alternatively, the new economics of labor migration (NELM) framework suggests that voluntary return migration is a product of successful migration (Cassarino 2004), predicting positive selection of returnees on economic predictors (de Haas et al. 2015). This framework holds that the decision to migrate occurs at the household level to diversify income sources and reduce family economic risk associated with limited credit and insurance in origin countries (Stark 1991). Immigrants often evaluate compensation, work conditions, and social mobility in terms of the standards in their origin country (Piore 1979), as Durand (2004:109) noted for Mexicans “earning in dollars, spending in pesos.” According to this model, voluntary return migration is one natural culmination of successful migration; immigrants return once they have accumulated sufficient assets (Kirdar 2009) or human capital (Dustmann et al. 2011) for household socioeconomic mobility in their origin country (Hagan et al. 2015). Lending empirical support for the NELM framework among Mexican returnees, studies have found a positive association between voluntary return migration and human capital (Hagan et al. 2015; Reinhold and Thom 2009), economic integration, and asset acquisition after returning to Mexico (Ambrosini and Peri 2012; Massey and Espinosa 1997; Massey and Parrado 1998). However, these studies did not contrast returnees with those staying in the destination country and did not examine total wealth.

### Differential Economic Considerations by Life Course Stage

The theories reviewed in the previous section relate to the general immigrant population and do not explicitly consider heterogeneity in economic selection by age at return. However, migration decisions are guided by life stage, and return migration is no exception (Jeffery and Murison 2011; Reagan and Olsen 2000). Overall, the probability of voluntary return migration is curvilinear by age, higher soon after migration at young ages and at retirement ages (Kirdar 2005; Massey et al. 2015; Van Hook and Zhang 2011; Vega and Brazil 2015).

Economic and labor force considerations differ by life stage, which may implicate variation in the economic selection mechanism for younger versus older returnees. Both younger and older returnees may be negatively selected on wealth. Hardship integrating into the U.S. labor market and the resulting limited wealth accrual may be associated with midlife return migration. Among older adults, as they approach exit from the labor force and anticipate greater dependence on assets, those with less accumulated wealth may be more likely to return in order to secure higher purchasing power in their origin country and the assurance of a higher standard of living (Dustmann 1997; Hill 1987; Stark et al. 1997). On the contrary, both younger and older returnees might be positively selected on wealth. Evidence from Europe suggests that immigrant savings rates decrease over time in the destination country. Therefore, immigrants who are economically successful may attain their asset goals within a few decades (Kirdar 2005), perhaps leading to positive selection on wealth for younger returnees. For older adults, return migration may indicate a wealth advantage if they return upon retirement or shortly thereafter. The same can be said for those at the boundary between younger and older adulthood, who might return before traditional retirement ages and forgo additional U.S. employment years at higher wages (Kirdar 2009). This logic follows the savings accumulation conjecture, which suggests that maximizing savings is often the motivation for migration, and return to maximize purchasing power is optimal once prospects for saving from higher incomes in the destination country decline (Kirdar 2009).

A few studies investigated the economic considerations for return migration at retirement age among Mexican immigrants in the United States. Vega (2015) found no association between Social Security benefit amounts and return migration and concluded that economic considerations might be a less salient motivation to return for older immigrants. Directly analyzing reported reasons to return among Mexican returnees, Vega and Hirschman (2019) found that economic reasons were not a principal motivating factor among older or younger returnees. In sum, theories on voluntary return migration leave considerable uncertainty about wealth differences between returnees and stayers, hampering hypothesis construction regarding the contribution of return migration to immigrant wealth stratification. Although there may be selection into return migration on net assets, the direction of selection is unclear and may operate differently for younger and older returnees.

Beyond differential selection into return migration, age at return also has implications for economic reintegration after returning. Whereas older returnees may have amassed most of their assets in the United States for use in Mexico, those returning at younger ages are more likely to reenter the labor market in Mexico, use the human capital they accumulated in the United States to achieve wage growth and occupational mobility back in Mexico, or use financial capital from the United States to invest in business ownership (Gutiérrez Vázquez 2019; Hagan et al. 2015; Massey and Parrado 1998; Parrado and Gutiérrez Vázquez 2016). Without differentiating life course timing of return migration, studies cannot consider variation in capital amassed in the United States versus Mexico and the underlying determinants of this variation.

To my knowledge, no empirical research has directly compared Mexican immigrants’ assets at older ages by return migration status or considered life stage at return as it relates to selection into return migration or trajectories of wealth accumulation. Given the nonnegligible portion of Mexican immigrants to the United States who ultimately return to Mexico (Azose and Raftery 2019), return migration must be considered as a factor contributing to wealth stratification among Mexican immigrants.

### Contribution

This paper contributes empirically by creating a novel data set representing Mexicans aged 50 and older with a history of migration to the United States to investigate return migration as a source of wealth stratification for Mexican immigrants in the United States. It first asks how wealth differs among stayers, returnees at younger ages, and returnees at older ages. It then asks how compositional differences between returnee groups explain or express asset accumulation by modeling total net wealth as a function of demographic, premigration, socioeconomic, and migration-related factors. Next, it details how variation in assets differ by age at survey, age at migration, and time since return (for returnees) by visualizing total net assets for all three groups on these factors. Finally, it explores heterogeneity in wealth outcomes within the population of stayers by evaluating correlates of zero wealth and debt relative to positive wealth. The results inform our theoretical understanding of mechanisms of economic selection into return migration by life stage, deepen our understanding of factors contributing to the low wealth attainment of Mexican immigrants in the United States, and challenge existing approaches to measuring immigrant socioeconomic attainment.

## Methods

### Data and Analytic Sample

Data for this analysis come from the Health and Retirement Study (HRS) and the Mexican Health and Aging Study (MHAS). The HRS is a nationally representative panel study of Americans older than 50. Data collection for the HRS began in 1992, with follow-up surveys every two years and a refreshment sample added every six years (HRS Staff 2008). The HRS was designed to follow the transition into retirement among the U.S. population and to understand the interactions among health, economics, and family structure at older ages (Servais 2010). The HRS oversamples Latinos^3^ and other populations at a rate of 2 to 1, increasing the original sample from 5% (based on CPS estimates) to 9% of the total HRS sample (Heeringa and Connor 1995).

The MHAS is a nationally representative panel study of Mexicans 50 and older. The baseline survey was conducted in 2001, with follow-up surveys in 2003, 2012, 2015, and 2018. Like the HRS, the MHAS uses a steady-state design, periodically adding new age-eligible samples (in 2012 and 2018). The MHAS was designed to understand aging in Mexico and is highly comparable with the HRS in study protocols and survey instruments (Wong et al. 2017). The 2001 MHAS oversampled households in the six Mexican states that accounted for 40% of U.S. migrants in 2001 (Wong et al. 2017). With weights, the MHAS data are nationally representative, providing rich data on Mexicans 50 and older with a migration history.

Following other studies combining the HRS and MHAS (Dĺaz-Venegas et al. 2016; Gerst et al. 2011; Monteverde et al. 2010), for temporally comparable samples across data sets, I combine the 2001 MHAS data with the 2000 HRS follow-up survey to create a weighted binational sample stratified by country, following guidelines from the Gateway for Global Aging for cross-national analysis using HRS sister studies. The result is a unique data set that reflects the middle-aged and older population in both the United States and Mexico. The analytic sample is limited to those aged 50 and older at the time of the survey^4^ who first immigrated to the United States from Mexico before age 50. The online appendix (section 1) details case selection. The final sample includes 1,392 Mexicans with an immigration history to the United States.

Missing data are handled using a dummy variable approach following research using similar variables from HRS (Tarraf et al. 2020). This approach, which includes a category for missing, preserves the already limited sample size. It accounts for potentially nonrandom missingness on covariates, an advantage over multiple imputation.

### Measures

HRS and MHAS ask questions about 20 categories of wealth and include data on detailed asset holdings. Total net wealth (total assets minus total debt), the main dependent variable, and component assets (net primary home, other real estate, business, financial, and vehicle assets) are taken directly from RAND HRS and the Harmonized MHAS data files. Additionally, I construct an indicator for positive assets and assets relative to Mexican and U.S. median wealth. Details on measurement, nonresponse, unfolding brackets, and imputation of wealth data are included in the online appendix (section 2).

Assets, collected at the household level, are adjusted for household size by dividing net wealth by the square root of total coresidents following previous cross-national research on wealth (Gornick, Sierminska et al. 2009).^5^ Data from MHAS, originally reported in pesos, are converted to dollars using purchasing power parities (PPP for private consumption; OECD 2019) and historical annual average exchange rates. PPP incorporates U.S.-Mexico differences in the cost of living that may be part of the decision to return.

The key predictors are a set of dummy variables representing returning versus staying in the United States by age at return: (1) *stayers*: Mexican immigrants from the HRS sample who remained in the United States past age 50; (2) *younger returnees*: Mexican immigrants to the United States from the MHAS sample who returned to Mexico before age 50; and (3) *older returnees*: Mexican immigrants to the United States from the MHAS sample who returned to Mexico at age 50 or older.

Informed by research on return migration and wealth across the life course, covariates include demographic variables (age in five-year age groups and indicators for female and married), premigration (an indicator for fair/poor childhood health), adult socioeconomic status (education measured as elementary school or less; less than high school; high school, vocational school, or some college; or college degree or more and harmonized for cross-national comparison by the Gateway for Global Aging), integration (continuous variable for years in the United States), and controls (indicators for urban U.S. residence and U.S. migration before age 18). In the online appendix, section 3 describes the construction of measures from original variables, and section 4 presents unweighted descriptive statistics and details missing values. I explored a more extensive set of potential confounders^6^ in supplementary analysis but include a limited set here to avoid multicollinearity and overfitting.

### Analytic Strategy

To account for survey design and oversampling, I conducted the analysis in Stata/MP 17.0 using the *svy* suite of commands and weighted the data.^7^ Weights were provided by the RAND HRS Longitudinal File and the Harmonized MHAS data file. I used these weights in combination, stratified by country, following examples of cross-national analyses provided by the Gateway for Global Aging. I use an adjusted Wald test to compare differences between returnee groups in total net assets, net component assets, positive assets, and assets relative to Mexican and U.S. median wealth. Total net worth is modeled as a function of returnee group, adjusting for compositional differences between returnee groups, using ordinary least-squares (OLS) regression.

Because wealth tends to be positively skewed, log or inverse hyperbolic sine (IHS) transformations are often used (Pence 2006). An IHS transformation was chosen over a log transformation because IHS does not require discarding nonpositive values, which is particularly important when modeling wealth where zeros (i.e., no wealth) and negative values (i.e., debt) are substantively meaningful. IHS transformations are not invariant to scaling; units of measurement impact unit-free regression results. Following Aihounton and Henningsen (2021) and Norton (2022), I use multiple diagnostic criteria to determine the appropriate scale and use Duan’s nonparametric smearing estimate (Duan 1983) for retransformation into the original scale to estimate marginal effects. See the online appendix (section 5) for details on IHS scaling and retransformation.

### Sensitivity Analyses

Several sensitivity analyses confirmed the robustness of results to alternate analytic decisions. Table A2 in the online appendix reports the results of the final nested OLS model predicting IHS transformed total net wealth (column 1) with sensitivity checks for the exclusion of respondents who entered the United States as children or young adults (column 2), the exclusion of respondents who spent less than two or three years in the United States (columns 3–4), the modification of *older returnees* to those older than 55 and older than 60 (columns 5–6), and predicting only *nonhousing* financial wealth^8^ (column 7).

The MHAS does not collect data on the value of retirement accounts. To create comparable measures of wealth across surveys, I do not include retirement assets in the outcome variable, total net wealth (see section 2, online appendix). Table A3 in the online appendix tests the sensitivity of results to this decision by including in the net assets outcome variable the retirement assets for stayers, which *are* measured in HRS.

I also analyzed more recent samples (2012, 2018) to substantiate the robustness of my findings (available upon request). I do not present the results here because of the MHAS sample design: when adding new cohorts of individuals that age into the original 2001 sample, MHAS does not refresh the sample of already age-eligible individuals, omitting immigrants who return to Mexico between samples.^9^

## Results

### Sample Characteristics

Table 1 presents descriptive statistics for the full sample, for stayers, and by returnee group, showing evidence of compositional differences between groups that represent divergent human capital and migration experiences. These differences have implications for asset accumulation in the United States and upon return. On average, stayers are younger (62.6 years) than both groups of returnees (65.2 for younger and 68.2 for older returnees), and a larger percentage of stayers are women (51%) compared with returnees (17% for younger returnees and 20% for older returnees). Compared with stayers, younger returnees have more bifurcated educational attainment, with a higher proportion having an elementary school education or less (55% for stayers vs. 66% for younger returnees), a lower proportion having a high school education (11% vs. 3%), and a higher proportion having a college degree (2% vs. 8%). Total years in the United States differ considerably between groups: on average, stayers were in the United States for a total of 37 years, compared with approximately three years for younger returnees and 19 years for older returnees. Unlike younger returnees, who spent limited time in the United States before returning, older returnees spent an average of two decades in the United States before returning. The three groups do not differ in age at first migration. However, returnees were less likely to live in U.S. urban areas relative to stayers. Upon return, older returnees were more likely to live in urban areas in Mexico than younger returnees. The three groups do not differ in era of first migration. Just over half arrived before 1965, when the Bracero Program brought Mexicans to the United States on temporary seasonal visas to work in agriculture. Approximately 5% arrived after the legislation of IRCA in 1986 heightened border enforcement, increased deportations, and deterred return migration among the undocumented (Massey et al. 2002).

### Patterns of Asset Accumulation

The bottom panel of Table 1 presents mean and median total net wealth for the full analytic sample and by stayer and returnee group using both PPP and exchange rate conversions of pesos to dollars. Comparing mean to median wealth, it is evident that total net assets have a considerable positive skew, indicating sizable levels of wealth inequality. This is particularly true for stayers, who have higher rates of debt and zero assets (5% and 8%, respectively) than younger (1% and 2%, respectively) and older (0% and 1%, respectively) returnees.

**Fig. 1:**
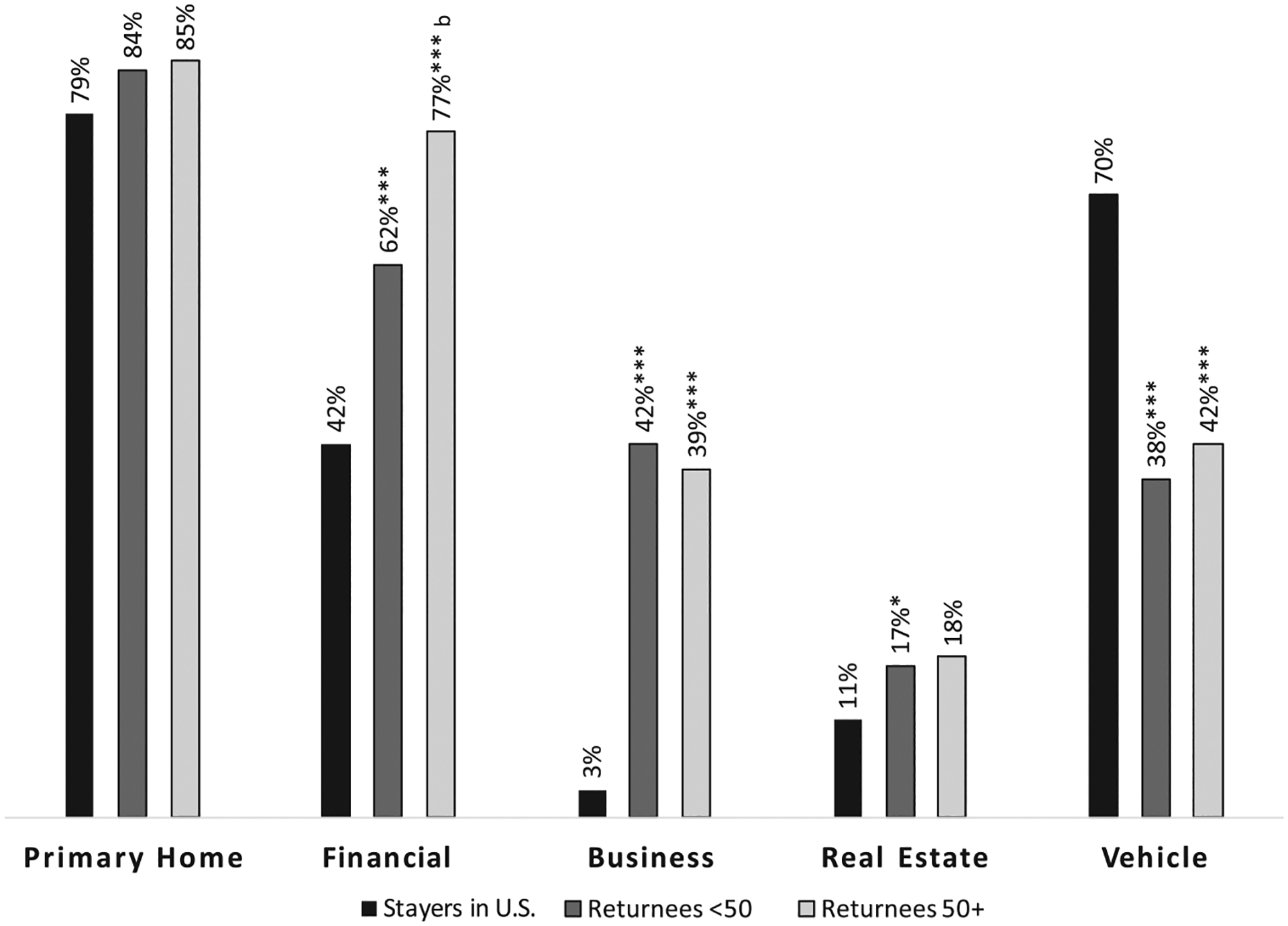
Positive assets holdings by asset type and Mexican immigrant stayer and return group. Asterisks indicate comparisons with stayers from an adjusted Wald test, and the superscript letter indicates comparison with returnees at age 50+ from an adjusted Wald test. *p < .05; ***p < .001 (compared with stayers). ^b^ *p* < .01 (compared with returnees at age 50+).

Among those with positive assets, the portion of each stayer and returnee group holding each wealth type is shown in Figure 1. Homeownership does not differ across groups and is the primary asset type. Among those with positive assets, more than three quarters of each group were homeowners. Younger returnees were more likely to hold real estate assets (i.e., a nonprimary home) than stayers (17% vs. 11%), with no statistically significant difference between older returnees and stayers or between the two returnee groups. Whether these investments were made before or after return is unclear. However, land and property investment in Mexico is one way immigrants prepare for returning, and investing in nonprimary homes may be less likely among those intending to remain in the United States.

Additionally, stayers were less likely to hold positive financial assets than returnees. Only 42% of stayers had financial assets, compared with 62% of younger returnees and 77% of older returnees. There are also considerable differences in the likelihood of holding business assets, with only 3% of stayers holding positive business assets, compared with approximately 40% of both returnee groups. Business ownership is an important source of wealth for returnees but is negligible for stayers. These are consequential differences indicating that stayers are less likely than returnees to hold positive assets and that those who do are less likely to have diverse asset holdings.

**Fig. 2:**
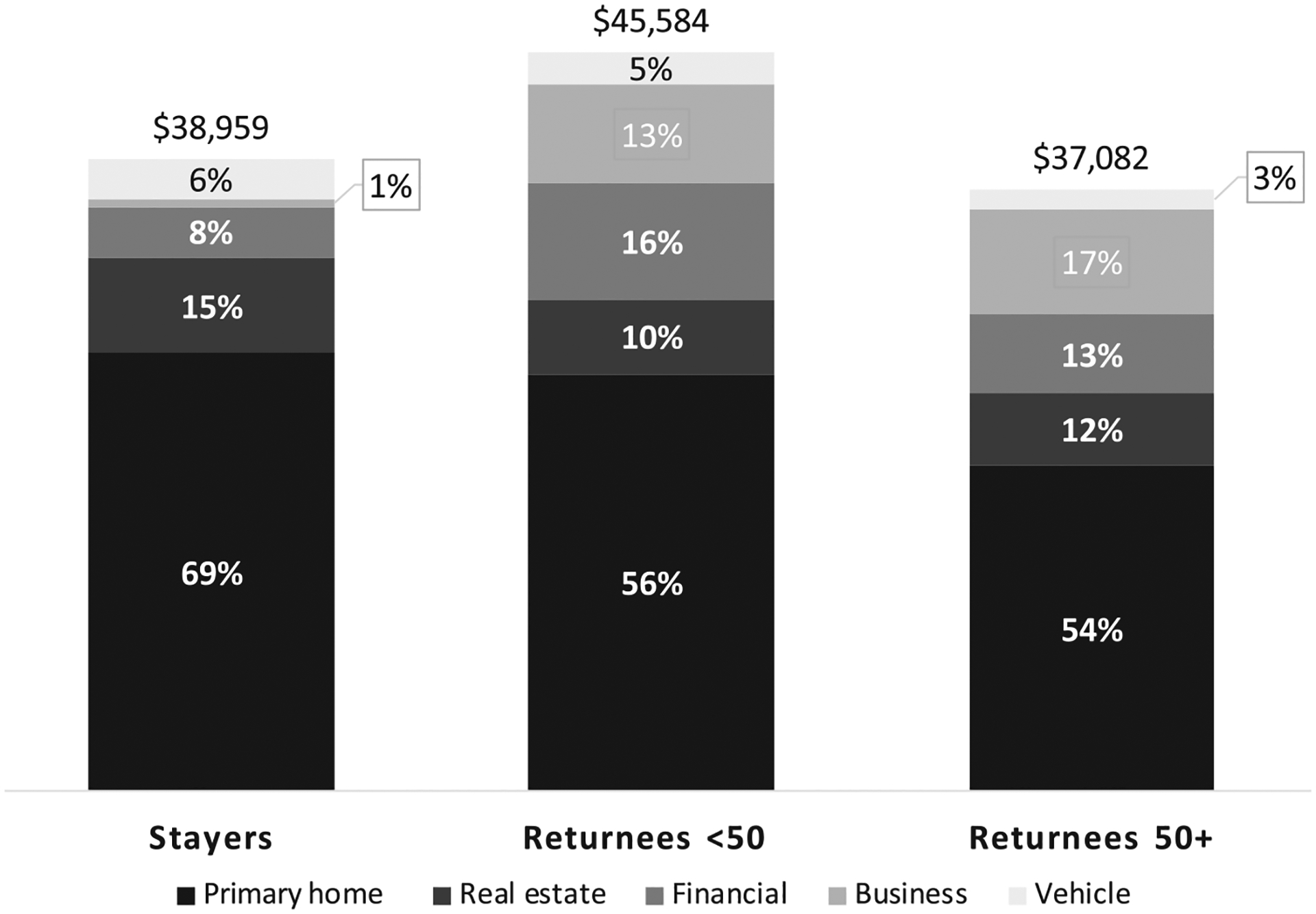
Mean total net assets and distribution of type by Mexican immigrant stayer and return group. Pesos are converted to U.S. dollars (2000) using PPP.

Figure 2 shows the portion of each component asset among total assets by stayer and returnee group. On average, 69% of stayers’ total assets were in homeownership and another 15% were in real estate. Younger and older returnees also had a sub-stantial portion of their total assets in homeownership (56% and 54%, respectively) and real estate (10% and 12%, respectively). Business and financial assets combined represent approximately 30% of the total assets of both groups of returnees. By contrast, these assets represent only 9% of stayers’ total assets. Thus, given that 13% of stayers do not have positive assets, stayers are quite dependent on real estate. Considering differences in assets across the three groups, this analysis points to wealth vulnerability among a portion of Mexican immigrants who remain in the United States past age 50.

### Purchasing Power and Relative Assets

Particularly at older ages, return migration is posited to be a means of maximizing savings through increased purchasing power. I quantify the increased purchasing power of assets spent in Mexico by comparing net worth using PPP to net worth using the standard annual average exchange rate (presented in Table 1). The purchasing power of the total assets of older returnees in Mexico is statistically equivalent to that of stayers in the United States. However, the exchange rate conversion, which represents the wealth older returnees would have if they had remained in the United States into older ages spending their assets in dollars, shows an advantage in estimated assets for stayers over older returnees. The advantage that purchasing power affords older returnees could point to a calculation among this group to return to Mexico to ensure a better standard of living using their assets as they approach retirement. There are no statistically significant differences in total net assets between younger returnees and stayers across conversion types.

**Fig. 3:**
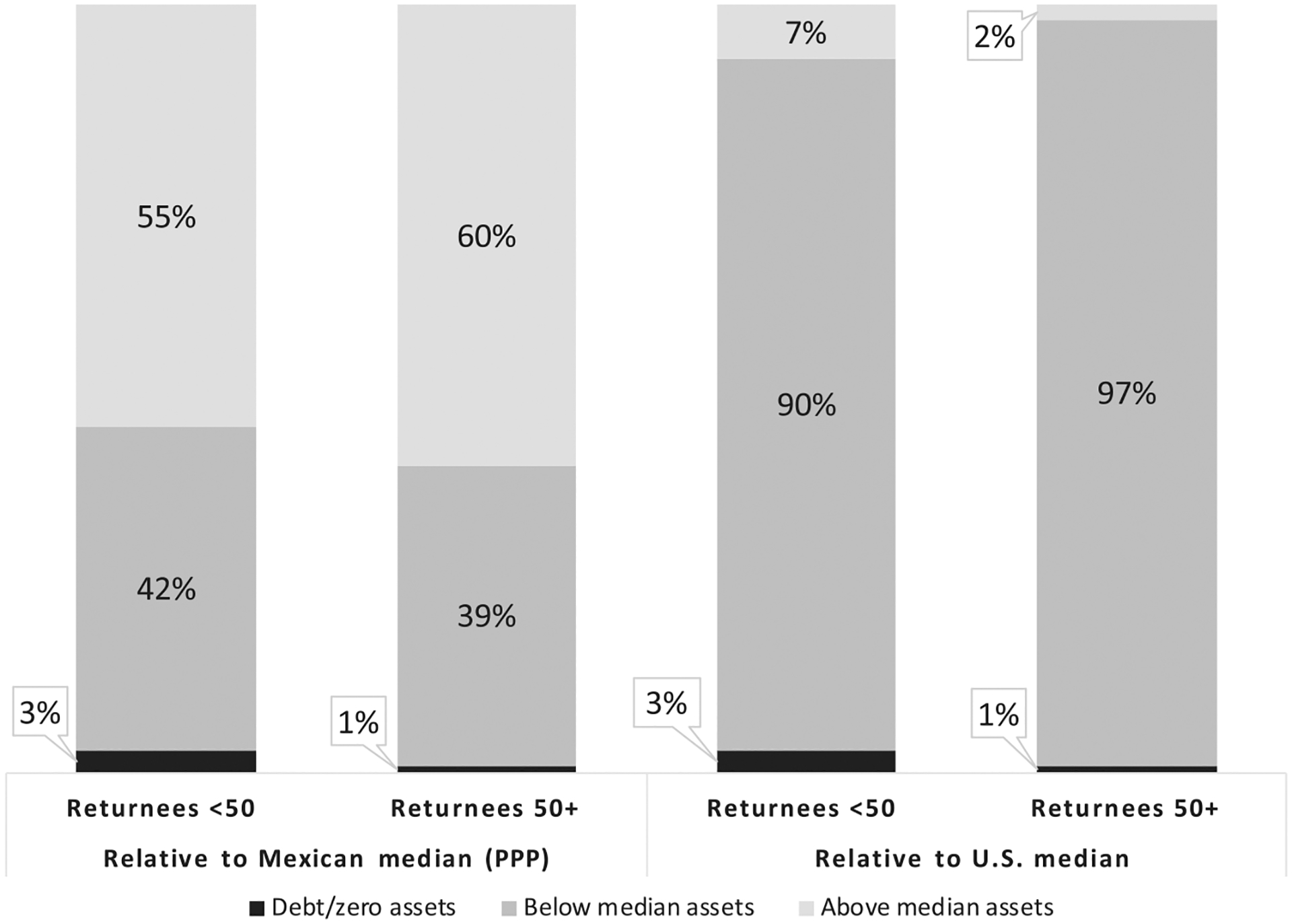
Assets of returnees relative to Mexican and U.S. (2000 exchange rate) median assets

The left half of Figure 3 compares returnees’ net assets to the median total net wealth of the entire MHAS sample of Mexican residents older than 50.^10^ More than half of each returnee group is estimated to have wealth higher than the Mexican median wealth. The right side of Figure 3 looks at the situation from the opposite direction. Returnees are less likely to have wealth above the U.S. median (calculated using the entire HRS sample of American residents older than 50).^11^ Among older returnees, 60% hold wealth higher than the Mexican median, but only 2% hold wealth higher than the U.S. median.

### Understanding Net Worth

**Fig. 4:**
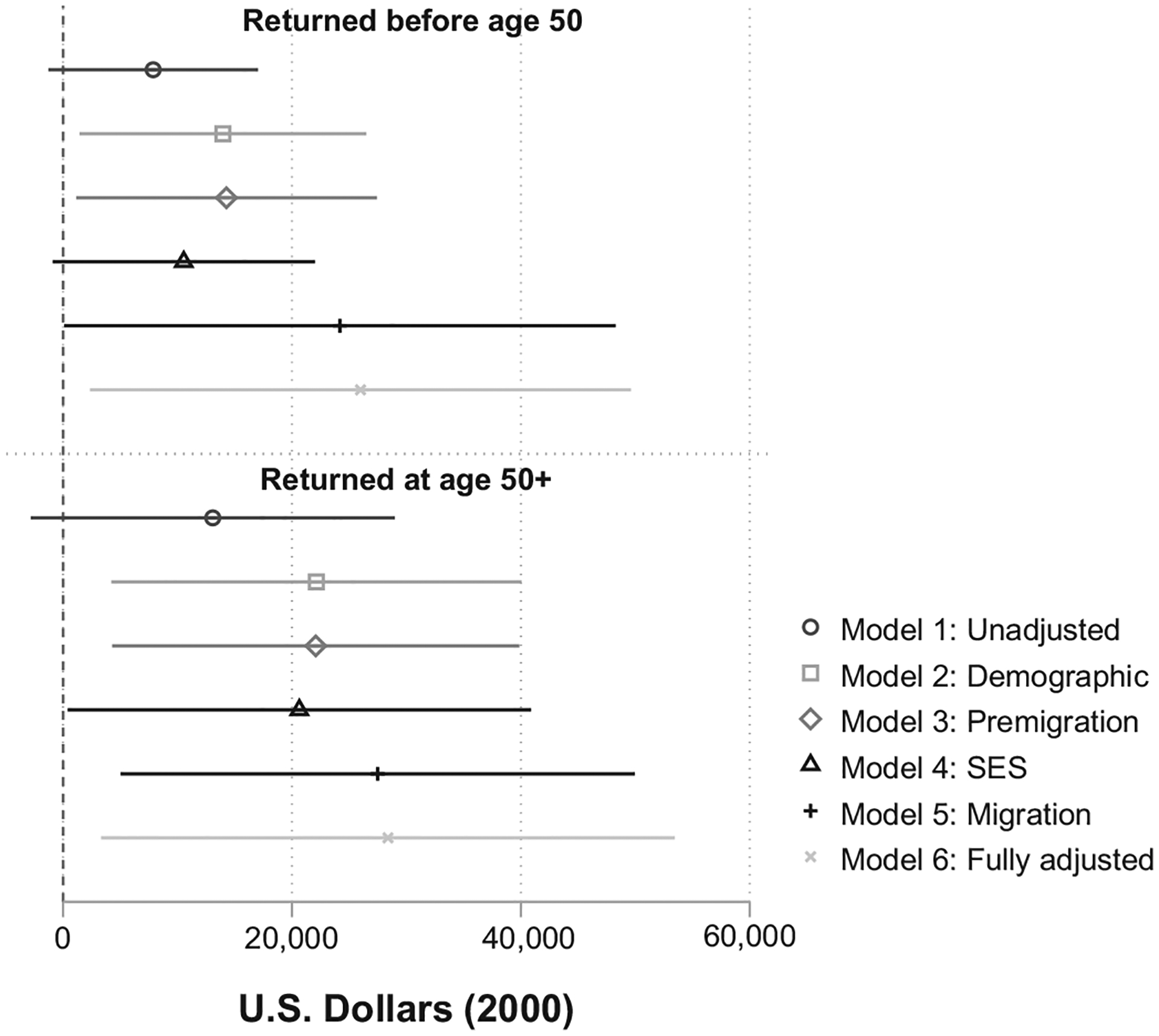
Average marginal effects of return group (ref. = stayers) on net total wealth (in 2000 US$), by model

Next, I model total net worth (in PPP), transformed with IHS, and scaled, by group. I use OLS regression with adjustments for demographic composition (age, gender, and marital status), a premigration characteristic (childhood health), socioeconomic status (education), and integration in the United States (years in the United States) included separately and then in a final fully adjusted model. All models except the first, unadjusted model include controls for urban residence in the United States and migration as a child to account for differences in return migration patterns and integration by settlement region and age at migration. Table 2 presents model coefficients, and Figure 4 presents average marginal effects by model on the original scale (2000 dollars) for both returnee groups relative to stayers.

Results from Model 1 are consistent with those from Table 1: without controls, all three groups hold statistically equivalent PPP adjusted net worth. Once adjustments for compositional differences in age, gender, and marital status and controls for U.S. urban residence and childhood migration are included in Model 2, both younger and older returnees hold a wealth advantage. Figure 4 shows that the wealth advantage over stayers is roughly $14,000 for younger returnees and $22,000 for older returnees. In Model 3, which adjusts for childhood health, the returnee advantage remains similar to that in Model 2. After controlling for educational attainment in Model 4, college has a large effect on wealth accumulation. Further, differences in educational attainment for returnees relative to stayers explain the younger returnee wealth advantage and reduce but do not eliminate the advantage of older returnees over stayers. Results from Model 5, which controls for years in the United States, show that both returnee groups retain their wealth advantage over stayers. Finally, both groups of returnees have more than a $25,000 wealth advantage over stayers once the full range of compositional differences are incorporated into Model 6 (see Figure 4). Supplementary analyses comparing the two returnee groups showed no statistically significant differences in wealth.

### Variation in Assets

**Fig. 5:**
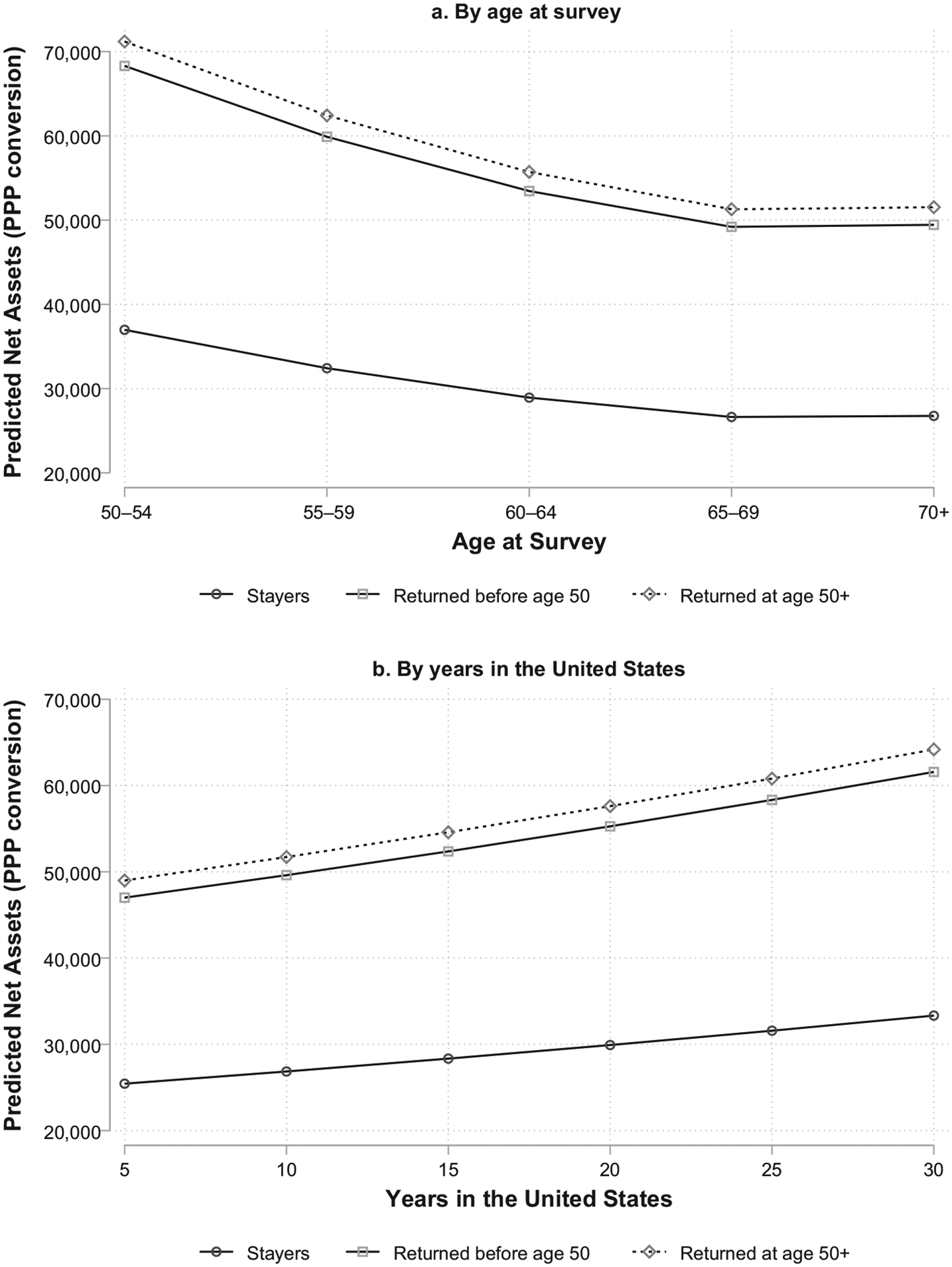

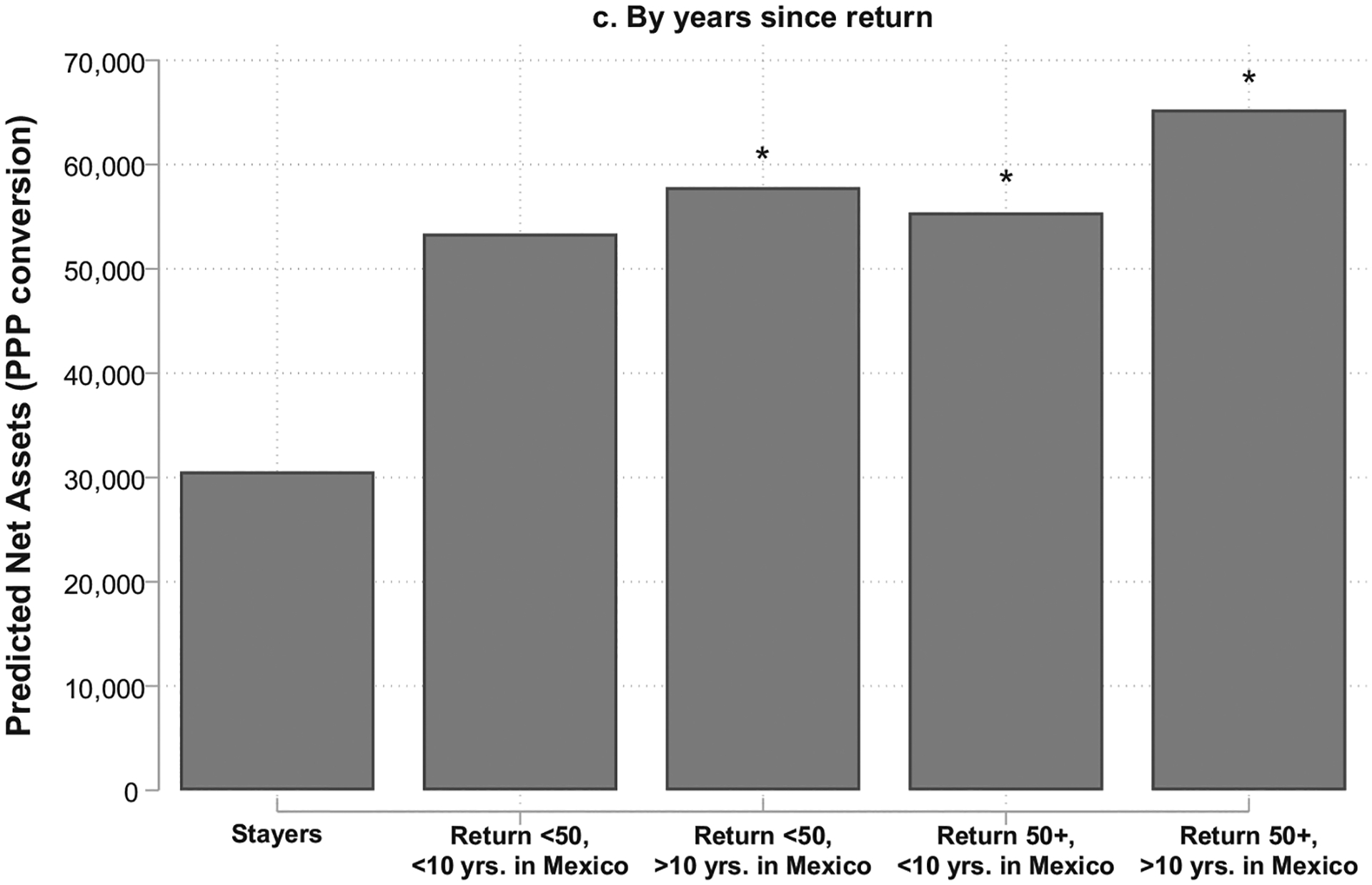
Variation in net total wealth (in 2000 US$) by age at survey, years in the United States, and years since return. Estimates are adjusted for all covariates in Model 6: age at survey, sex, married, fair/poor childhood health, education, years in the United States, migration before age 18, and U.S. urban residence. In panel *c*, *p < .05 (ref. = stayers).

Asset accumulation may vary by age at survey and years in the United States. To investigate these patterns, I plot in Figure 5 the estimates of net worth from the fully adjusted model by age at survey (panel a) and years in the United States (panel b). Panel a shows that net assets decline with age for all three groups, but differences between groups remain consistent. Similarly, panel b illustrates the positive association between years in the United States and wealth for all three groups. Interactions of group with age at survey and with years in the United States were considered in auxiliary analyses but were not jointly significant.

Additionally, there may be variation within both groups of returnees by years since return to Mexico. Panel c of Figure 5 plots estimated net wealth from the fully adjusted model, dividing the two returnee groups into those who returned to Mexico within the last 10 years and those who returned more than 10 years ago. Statistical significance should be considered with caution because of small sample sizes, especially for younger returnees within 10 years of return (*n* = 50) and older returnees who returned more than 10 years ago (*n* = 63). With that in mind, I find that all returnees except older returnees who returned more than 10 years ago are estimated to hold higher adjusted net worth than stayers.

### Correlates of Wealth Among Stayers

The preceding results show evidence of a wealth advantage for returnees relative to stayers, especially after I adjust for compositional differences and correlates of return migration and asset accumulation. However, to conclude that stayers as a whole are more vulnerable to limited assets at older ages than returnees misses the heterogeneity evidenced by their skewed wealth distribution. To examine the profile of stayers who are especially vulnerable to wealth insecurity, Table 3 presents the distribution of stayers by wealth category (debt, zero assets, and positive assets) and key characteristics. Debt and zero assets are separated because debt (i.e., negative assets) may indicate borrowing capacity and the process of building assets, whereas zero assets represent the least borrowing power (Hao 2004). In fact, Flippen (2020) found that holding debt with formal institutions is associated with financial incorporation rather than distress. Although theoretically meaningful, this division results in small sample sizes for those in debt and with zero wealth, so estimates should be interpreted with caution.

As evident from Table 3, those with zero wealth and debt differ substantively from those with positive wealth in a couple of ways. On average, relative to stayers with positive wealth, stayers with zero assets are older and are less educated. Stayers with debt do not differ on these characteristics from stayers holding positive assets. Those with debt and zero wealth diverge from those with positive wealth in that they were older when they first migrated to the United States and are less likely to be married. Overall, older immigrants with zero wealth represent a particularly precarious group of older Mexican immigrants in the United States.

## Discussion

To investigate the links between wealth and return migration for Mexican immigrants to the United States, this analysis considers the net wealth at age 50 and older of three groups of Mexicans with an immigration history: stayers, younger returnees, and older returnees. Across the life course, return migration is associated with a more diverse asset portfolio, a lower probability of debt or zero wealth, and (after compositional differences between stayers and returnees are accounted for) higher total net wealth relative to remaining in the United States into older ages. Additionally, a portion of stayers—those who are older, unmarried, and migrated at older ages—are particularly vulnerable to asset insecurity at older ages.

This analysis provides empirical evidence that selective return migration may be one driver of nativity-based wealth stratification in the United States. Financial dependence on family in the United States or the cost of returning and reestablishment may preclude return migration for immigrants with lower assets, particularly those with debt or zero assets, leaving a residual population in the United States with limited assets. Just as selective return migration among immigrants in worse health is one factor contributing to an immigrant mortality advantage for stayers relative to the U.S.-born population (Turra and Elo 2008), selective return migration among immigrants with more wealth may contribute to the wealth disadvantage of stayers relative to the U.S.-born population.

This study also questions conclusions about Mexican immigrant socioeconomic mobility based on empirical findings solely considering immigrants remaining in the United States. Asset accumulation is a key measure of socioeconomic mobility for immigrants because it reflects processes of immigrant incorporation (Keister et al. 2015), and its use in the origin country has long been a primary goal of immigrants (Alba and Nee 2003). The results of this analysis show a more comprehensive picture of the economic outcomes of the full population of Mexican immigrants with a history of migration to the United States. The wealth advantage of returnees indicates that evaluating the socioeconomic success of immigrants without considering returnees downwardly biases estimates, yielding an underestimate of Mexican immigrants’ socioeconomic mobility. For older returnees, I find evidence that return migration is undertaken to ensure socioeconomic mobility via increased purchasing power.

Theoretically, this analysis makes several contributions. First, it supports the NELM understanding of voluntary return migration. Return migration is associated with equivalent (unadjusted) or more (adjusted) assets when measured at age 50 and older. The finding that business assets represent a considerable portion of total assets for both returnee groups but are negligible among stayers supports the NELM conjecture that migration is used to accumulate assets for reinvesting back at home (Wassink and Hagan 2018). Because this analysis examines assets at age 50 and older, it offers support for the NELM perspective not at the point of return but over the long term. Although the available data do not include information about where assets were accumulated, these assets are likely not independent of the migration experience, even if younger returnees accumulated their assets upon returning to Mexico. Previous research shows that human capital gained in the United States leads to wage growth and occupational mobility after immigrants return to Mexico (Hagan et al. 2015). Therefore, asset accumulation of those who returned at younger ages can be considered an indirect result of migration. Moreover, these returnees did not re-emigrate from Mexico to the United States (re-emigrating was a common occurrence at the time) (Massey 1987). This finding points to a potential calculation among returnees that remaining in Mexico would lead to greater wealth accumulation in older adulthood.

This research also deepens our theoretical understanding of return migration at older ages specifically. Here, comparing assets converted from pesos to dollars using PPP (the value of money in Mexico) versus exchange rates (the value of money in the United States) indicates that for older returnees, return migration nonnegligibly increases the spending power of their assets. This result confirms Kirdar’s (2009) savings accumulation conjecture that higher PPP in the origin country is a more dominant force for older adults who, by returning, are not giving up as many years of higher wages in the destination country. For older returnees, return migration can be understood as a financial strategy undertaken upon approaching or entering retirement to maximize the value of assets and ensure a higher standard of living and housing conditions in retirement.

The data used in this study preclude an analysis of wealth and return migration after 2001, and findings may not hold for contemporary returnees. In fact, research on more recent patterns of economic reintegration of returnees to Mexico shows that the increase in deportations since 1986 and the 2007 recession in the United States resulted in compositional changes to returnees themselves and the economic and geographical resettlement decisions they make upon return. Compared with the preceding two decades, by 2010, returnees experienced reduced earnings, which have been partially attributed to a lower likelihood of engagement in entrepreneurial activities, a shift to entering the paid labor force with employment in the informal economy or occupations that lack benefits, and less positive selection on educational attainment (Campos-Vazquez and Lara 2012; Gutiérrez Vázquez 2019; Parrado and Gutierrez Vazquez 2016). Additionally, Denier and Masferrer (2020) showed that geographical changes in patterns of returnee settlement from 2000 to 2015 are also associated with decreased earnings. They found that increasing numbers of recent returnees settle in nontraditional migration-sending regions in northern, southern/southeastern, and central Mexico, where wages in this period declined the most. The more involuntary nature of return migration and resulting changes in educational selectivity, employment patterns, and settlement may have important consequences for asset accumulation between return migration and older adulthood. Therefore, the comparative wealth of returnees and stayers may have changed since 2000, altering the implications of return migration on Mexican immigrant wealth stratification in the United States. This possibility should be analyzed when recent, nationally representative binational data are available.

Several limitations characterize this analysis. First, the HRS and MHAS do not provide data on immigrant legal status, and the MHAS does not distinguish between voluntary returnees and deportees. Therefore, this research does not assess the financial implications at older ages of undocumented status or heightened deportation policies (Masferrer and Roberts 2012; Riosmena and Massey 2012; Roberts et al. 2017). Second, the data preclude accounting for varying economic and structural factors by U.S. settlement region that may result in differing probabilities of return migration (Chort and de la Rupelle 2016; Lindstrom and Massey 1994). Likewise, because data on resettlement region upon return to Mexico are unavailable, the analysis does not account for geographical variation in economic opportunities for wealth accumulation (Denier and Masferrer 2020) between return migration and survey measurement. Third, the absence of immigration-specific life history data means that models estimated here cannot account for censoring; those identified as stayers may end up returning to Mexico later, and those identified as returnees may take an additional trip to the United States. However, because the sample is older-age individuals, the likelihood of additional migrations is limited. Fourth, for immigrants, wealth is often held abroad (Akresh 2011; Flippen 2020) or transferred to family transnationally (Garip 2012). However, neither the HRS nor the MHAS survey explicitly asks respondents to include or exclude assets held abroad when reporting asset amounts or to report transnational household transfers of wealth. This lack of specificity may be a source of wealth reporting error. Fifth, retirement assets are not included in the main results because they are not collected in MHAS, which could be a source of error. However, results from a sensitivity check (see Table A3, online appendix) show that the main finding of this analysis—a wealth advantage for returnees—is robust to this source of error.

Finally, because some unobserved characteristics that promote wealth accumulation may be the same as those that determine return to Mexico, the results could be impacted by omitted variable bias. Although modeling these factors is outside the scope of this descriptive paper, saving for investments in Mexico (e.g., business, real estate, education of children left in Mexico) during U.S. tenure might be associated with a higher likelihood of returning to Mexico. Legal status and health may also be factors associated with wealth accumulation and return migration, although the direction of bias in these cases is less clear. For example, evidence suggests that undocumented immigrants have lower wages (Hall et al. 2010; Massey and Gentsch 2014) and may be able to save less. Undocumented immigrants are more likely to be deported but may be less likely to return voluntarily (or engage in circular migration) than authorized immigrants because the cost and risk of unauthorized migration have increased since 1986 (Massey et al. 2015). Although this analysis does not discern the causal mechanisms, it provides a descriptive basis for future research on wealth and return migration.

This article makes important contributions to our understanding of Mexican immigrant wealth stratification in the United States. First, it indicates that selective return migration, regardless of the reasons for it, could be a driver of stratification. Net of compositional differences, returnees in the twentieth century held more wealth at older ages than stayers, leaving a residual population of Mexican immigrants in the United States with lower average net wealth than the total population of Mexicans with a history of U.S. migration. Second, this analysis shows that conclusions about Mexican immigrant socioeconomic mobility that omit returnees systematically exclude a group of immigrants with largely positive wealth outcomes. In assessing immigrant socioeconomic success, this population should not be discarded. Third, this article points to heterogeneity within the populations of returnees and stayers, which is important to consider in future work on the determinants of Mexican immigrant economic well-being. Finally, combining theories on the economics of labor migration vis-à-vis voluntary return migration with theories of life course sociology and retirement-age decision-making demonstrates that voluntary return migration is not an instance of failed migration but rather a story of success. This article lays the groundwork for future research on differential selection into return migration by age and its implications for immigrant integration, stratification, and mobility among the Mexican immigrant population to the United States and other immigrant populations worldwide.

## Supplementary Material

supp

## Figures and Tables

**Table 1:** Sample characteristics by return group (weighted): HRS 2000/MHAS 2001

	Full Sample		Stayers		Returnees <50		Returnees 50+	
	Mean	SE	Mean	SE	Mean	SE	Mean	SE
Demographic Characteristics								
Age	64.26	0.38	62.56	0.58	65.20***	0.50	68.23***^b^	1.02
Female	0.33	0.02	0.51	0.04	0.17***	0.02	0.20***	0.06
Married	0.73	0.02	0.71	0.03	0.75	0.02	0.72	0.06
Premigration Characteristics Fair/poor childhood health	0.14	0.01	0.08	0.02	0.18***	0.02	0.20	0.06
Adult Socioeconomic Status								
Elementary school or less	0.61	0.02	0.55	0.04	0.66*	0.03	0.69	0.07
Less than high school	0.27	0.02	0.32	0.03	0.23*	0.02	0.20	0.06
High school, vocational school, or some college	0.07	0.01	0.11	0.02	0.03***	0.01	0.02***	0.01
College degree or more	0.05	0.01	0.02	0.01	0.08**	0.02	0.09	0.04
Migration								
Total years in United States	19.99	0.78	36.90	0.91	3.29***	0.20	18.98***^a^	2.16
Age at first migration	26.12	0.46	26.11	0.83	25.93	0.49	27.12	1.51
Migrated as a child	0.21	0.02	0.23	0.03	0.19	0.02	0.18	0.05
Years since return to Mexico	—	—	—	—	34.21	0.67	10.44^a^	1.09
Urban U.S. residence	0.63	0.02	0.88	0.02	0.40***	0.03	0.52***	0.06
Urban residence in Mexico at return	—	—	—	—	0.51	0.03	0.63^c^	0.05
Period of first migration								
Before 1965	0.53	0.04	0.53	0.04	0.59	0.03	0.63	0.05
1965–1985	0.43	0.04	0.43	0.04	0.36	0.03	0.30*	0.05
1986 onward	0.04	0.02	0.04	0.02	0.05	0.01	0.07	0.02
Wealth								
Purchasing power parity conversion (2000 US$)								
Mean total net assets	41,865	4,246	38,959	5,410	45,584	7,418	37,082	4,318
Median total net assets	20,160	1,364	21,567	2,846	18,704	1,514	25,343	5,465
Exchange rate conversion (2000 US$)								
Mean total net assets	34,970	3,489	38,959	5,410	32,539	5,295	26,470*	3,082
Median total net assets	16,942	1,278	21,567	2,846	13,352**	1,081	18,090	3,901
Distribution of debt, zero, and positive assets								
Debt	0.02	0.01	0.05	0.01	0.01**	0.00	0.00**	0.00
Zero assets	0.05	0.01	0.08	0.02	0.02**	0.00	0.01***	0.01
Positive net assets	0.93	0.01	0.87	0.02	0.97***	0.01	0.99***	0.01
*N*	1,392		321		903		168	

*Notes*: Asterisks indicate comparisons with stayers from an adjusted Wald test. Superscript letters indicate comparisons with returnees at age 50+ from an adjusted Wald test.

* *p* < .05;

** *p* < .01;

*** p < .001 (compared with stayers)

a *p* < .05;

b *p* < .01;

c p < .001 (compared with returnees at age 50+)

**Table 2:** OLS regression coefficients predicting IHS-transformed total net wealth (weighted)

	Unadjusted (1)	Demographic (2)	Premigration (3)	Socioeconomic Status (4)	Migration (5)	Fully Adjust ed (6)
Return Group (ref. = stayers)						
Return before age 50	0.25 (0.15)	0.38* (0.17)	0.39* (0.18)	0.26 (0.14)	0.64* (0.30)	0.61* (0.27)
Return at age 50 or older	0.39 (0.22)	0.55** (0.20)	0.55** (0.20)	0.45* (0.20)	0.70** (0.27)	0.65* (0.27)
Migrated Before Age 19		0.61*** (0.15)	0.60*** (0.15)	0.48** (0.15)	0.51** (0.16)	0.34* (0.16)
Urban U.S. residence		0.33* (0.16)	0.33* (0.16)	0.08 (0.14)	0.29 (0.17)	0.04 (0.14)
Missing urban U.S. residence		0.97*** (0.20)	0.97*** (0.20)	0.37 (0.48)	0.89*** (0.24)	0.25 (0.54)
Age (ref. = 50–54)						
55–59		−0.11 (0.23)	−0.11 (0.23)	−0.15 (0.21)	−0.10 (0.22)	−0.13 (0.21)
60–64		−0.22 (0.20)	−0.22 (0.20)	−0.23 (0.20)	−0.24 (0.20)	−0.25 (0.20)
65–69		−0.27 (0.24)	−0.26 (0.24)	−0.28 (0.21)	−0.30 (0.24)	−0.33 (0.21)
70+		−0.27 (0.19)	−0.26 (0.19)	−0.24 (0.19)	−0.33 (0.21)	−0.32 (0.20)
Female		−0.08 (0.19)	−0.07 (0.19)	−0.12 (0.17)	−0.06 (0.19)	−0.09 (0.18)
Married		0.67*** (0.20)	0.67*** (0.20)	0.67*** (0.18)	0.68*** (0.20)	0.68*** (0.18)
Fair/Poor Childhood						
Health			−0.09 (0.20)			−0.05 (0.19)
Missing childhood health			0.43 (0.35)			0.45 (0.36)
Education (ref. = elementary or less)						
Less than high school				0.30* (0.15)		0.30 (0.15)
High school, vocational school, or some college				0.87** (0.27)		0.89** (0.27)
College degree or more				1.87*** (0.47)		1.89*** (0.47)
Total Years in United States					0.01 (0.01)	0.01 (0.01)
Constant	3.20*** (0.13)	2.48*** (0.33)	2.48*** (0.33)	2.51*** (0.31)	2.24*** (0.42)	2.19*** (0.38)

*Note*: Standard errors are shown in parentheses.

* *p<*.05;

** *p<*.01;

*** *p*<.001

**Table 3:** Stayers by wealth category and key characteristics (weighted): HRS 2000/MHAS 2001

	Positive Net Assets		Debt		Zero Assets	
	Mean	SE	Mean	SE	Mean	SE
Age	62.14	0.62	62.61	1.38	67.21*	2.21
Female	0.49	0.04	0.61	0.14	0.69	0.10
Years of Education	5.35	0.34	4.70	0.89	3.85*	0.58
Married	0.76	0.04	0.37**	0.15	0.39**	0.12
Years in the United States	37.29	1.01	32.87*	1.57	35.26	2.85
Age at First Migration	25.28	0.91	30.46*	2.16	32.41***	1.74
*N*	276		15		30	

*Notes*: Asterisks indicate comparisons with those with positive net assets from an adjusted Wald test.

* *p* < .05;

** *p* < .01;

*** *p* < .001 (compared with those with positive net assets)

## Footnotes

1 Few studies of migration have simultaneously examined origin and destination in a way that enables contrasting returnees and those who remain at destination. The most prominent exceptions have focused on internal migration (e.g., Curran et al. 2016) or used qualitative or other non-population-based samples (Lubbers et al. 2020).

2 Even since the sharp increase in deportations after U.S. legislation in 1986 (IRCA) and 1996 (IIRIRA) (Massey et al. 2002) and the heightening of deportations following 9/11 (Golash-Boza 2015), most returnees have been voluntary (Passel et al. 2012).

3 Approximately 58% of Latino households in the HRS are of Mexican origin (Heeringa and Connor 1995).

4 The HRS and MHAS survey respondents’ age-eligible partners independent of age.

5 Gornick, Munzi et al. (2009) noted that taking the square root (or equivalence elasticity of 0.5) of household residents sets the adjustment between two theoretical possibilities: no economies of scale within the household and perfect economies of scale. Supplementary analyses compared this adjustment with dividing joint assets by 2 for married individuals (DeGraff et al. 2018; Purcell 2012) and found no substantive differences.

6 The additional confounders investigated in supplementary analyses were quadratic term for age, parental educational attainment, household size, year of first migration, age at first migration, citizenship status, adult self-rated health, health insurance coverage, and proximity to children.

7 I use household weights to estimate household-level assets and individual-level weights to estimate individual-level outcomes.

8 Real estate represents the largest share of assets for all three groups. Further, research on survey measurement of wealth has found that homeowners are, on average, quite accurate in reporting their home’s value relative to the actual value (Bound et al. 2001). However, this sensitivity check aims to assess the robustness of my findings to potential inaccuracies in reporting of real estate assets.

9 For example, in 2012, the MHAS added to the sample those individuals born in 1952–1962, who were not previously age-eligible for MHAS in 2001. However, it did not refresh the sample born in 1951 or earlier, thereby omitting from the 2012 sample those older than 60 who returned between sample years. This omission is particularly problematic because Mexican immigrants impacted by the subprime mortgage crisis and housing market crash in 2008, which disproportionately affected Latinos (Rugh 2015), would be included in the sample of stayers from the HRS but not in the sample of returnees from the MHAS.

10 This comparison converts pesos to dollars using PPP to evaluate how assets could be used in the Mexican economy relative to Mexican residents.

11 This comparison converts pesos to dollars using the average annual exchange rate to evaluate how assets could be used in the U.S. economy relative to American residents.
