# Supplementary material for: Immigrant Wealth Stratification and Return Migration: The Case of Mexican Immigrants in the United States During the Twentieth Century
Source: Demography. Author manuscript; Available in PMC 2023 Oct 16. (PMC10578872; doi:10.1215/00703370-10693686)
Supplement: supp [file NIHMS1936131-supplement-supp.pdf]

Online Appendix

"Immigrant Wealth Stratification and Return Migration: The Case of Mexican Immigrants in the United States During the Twentieth Century"

Mara Getz Sheftel

Section 1. Sample Selection – HRS 2000/MHAS 2001

The analytic sample excludes those less than 50 at the time of the survey[1]. It also excludes those who first immigrated to the US from Mexico after age 50 because older age migration is characterized by distinct motivating factors and integration processes (Litwak & Longino Jr, 1987). Cases with missing migration history data were also excluded: missing age/year of first migration (HRS/MHAS) and/or age/year of most recent return (MHAS). Four additional cases are excluded because they are missing across asset questions.

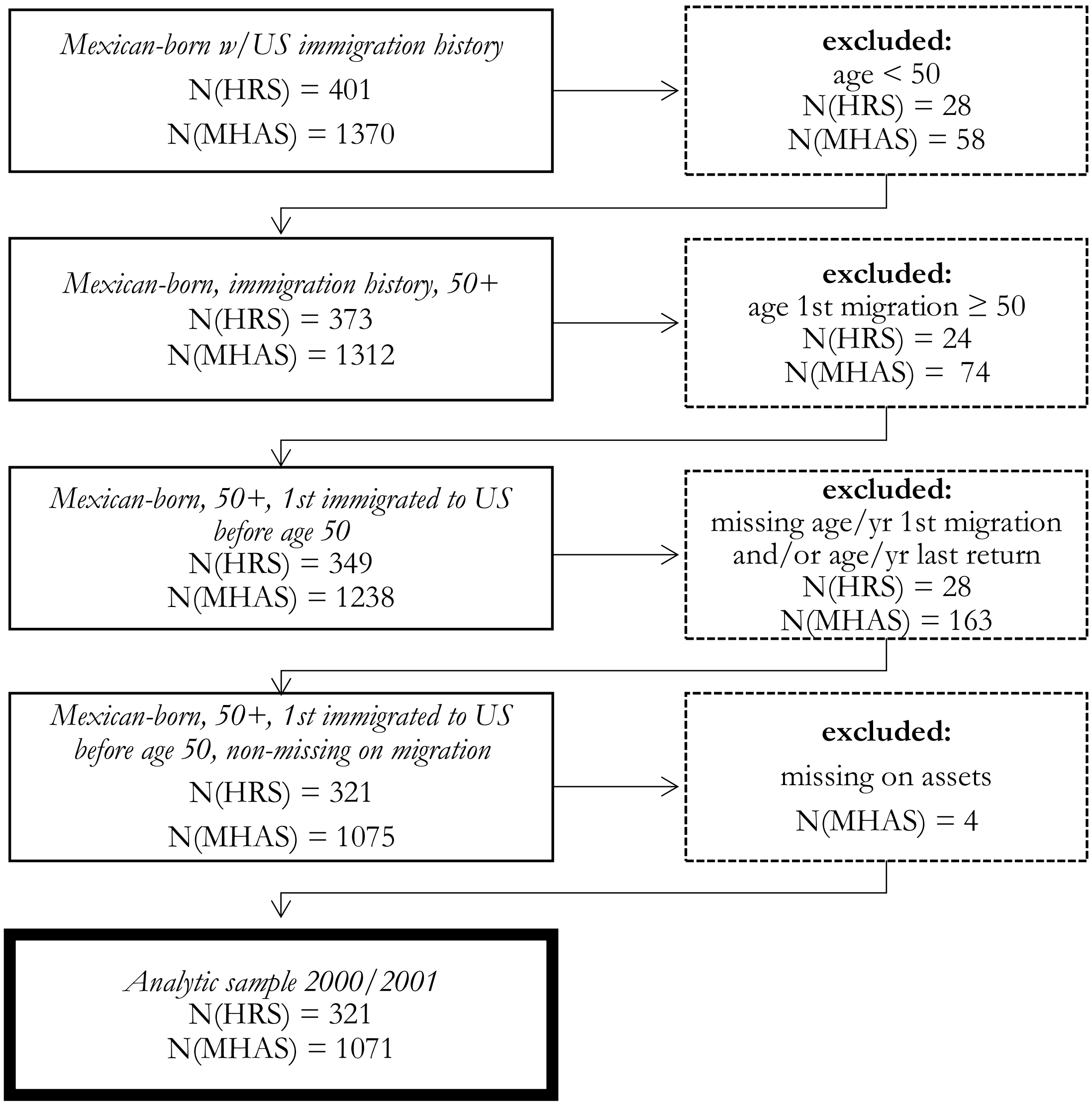

[1]HRS and MHAS survey partners of age eligible respondents independent of age.

Section 2. Wealth: measurement, validation, non-response, and comparability across surveys

HRS is the leading source of longitudinal survey data on the economic well-being of the older adult population in the US and has been at the forefront of methodological innovations in economic measurement (Hurd et al., 2016). MHAS stands out as unusual for providing detailed wealth information from a developing country (Wong & DeGraff, 2009). MHAS harmonized wealth variables were designed to be highly comparable to HRS despite crossnational differences in macroeconomic conditions, institutional differences in financial markets, and distinct individual savings habits (Angrisani & Lee, 2011; Michaels-Obregon et al., 2022). The outcome measures of wealth from HRS and MHAS are of the highest quality and have been internally and externally validated (Bosworth & Smart, 2009; Hurd et al., 2016; Keister, 2011; Sierminska et al., 2008; Wong & Espinoza, 2002).

Fries et al., (1998) outline three main reasons that survey-based measurement of household wealth is difficult. The following details how HRS and MHAS deal with these measurement challenges. First, rates of non-response tend to be higher for economic questions compared to other domains. This is particularly true of the highest income and wealth brackets. This means that survey instruments measure wealth, address initial non-response, and impute missing data must be sophisticated in order to produce reliable estimates. Below sections A and C detail measurement of wealth and handling of non-response in HRS and MHAS. Second, survey respondents may inadvertently report inaccurate values of assets and debt. This means that validation of survey data is necessary to ensure it is reliable. Section B details the validation of HRS and MHAS. Third, due to the high concentration of asset ownership, conventional methods of random sampling do not contain sufficient numbers of wealthy households to provide an accurate representation of the full distribution of wealth. Since this paper is not attempting to estimate distributional outcomes, this challenge is not a concern here. Crossnational comparisons of wealth bring in additional challenges due to differences in financial markets, investment products, and savings habits (Angrisani & Lee, 2011). Section D details the comparability of wealth measures between HRS and MHAS.

A. Measurement

Both HRS and MHAS ask survey respondents questions about 20 categories of wealth as described by Hurd et al. (2016) and Wong and Espinoza (2002). RAND HRS and the

Harmonized MHAS provides variables measuring net assets (total assets minus total debt) by type (primary home, other real estate, business, financial, and vehicle) and an overall measure composite measure of total net assets. Besides adjustments for household size, inflation, and exchange/purchasing power parity, the measures used in this paper are taken directly from the RAND HRS and the Harmonized MHAS datasets that have been processed for harmonization.

The only exception is the following:

- The Harmonized MHAS constructed total wealth composite measure (HwATOTB) includes real estate other than primary residence but not individual retirement accounts
- The RAND HRS wealth variable with the same name (HwATOTB) includes real estate other than primary residence AND individual retirement accounts.
- This discrepancy is because MHAS does not collect information about the amount in individual retirement accounts.
- Therefore, I use the RAND HRS HwTOTB and subtract the amount reported in individual retirement accounts to create a comparable total wealth measure.

B. Validation

The wealth measures included in both HRS and MHAS have been internally and externally validated.

a. HRS

Internally, HRS uses a cross-wave asset verification method to correct or confirm previous or current reports of assets if cross-wave differences are considerable (Hurd et al., 2016). In evaluating their asset verification HRS reports that "incorporating the asset verification data has small and nonsystematic effects on means of total household wealth and wealth components, but due to correction of a few large outliers standard deviations in some waves are reduced substantially," (Hurd et al., 2016, p. 5). Externally, HRS wealth measures have been validated using the Survey of Consumer Finance (Keister, 2011; Sierminska et al., 2008), the gold standard of data on household level wealth in the US (Gibson-Davis & Percheski, 2018; Keister, 2014; Kennickell, 2008; Wolff, 2016) as well as validated using comparisons to

macroeconomic data (Bosworth & Smart, 2009). The latter shows that HRS remains representative of the underlying population even considering its longitudinal structure and attrition over time.

b. MHAS

Wong and Espinoza (2002) detail the internal validation of MHAS wealth data showing that asset variables in MHAS reflect the expected patterns of wealth, for example a "highly skewed distribution in particular across education, and better economic position for unmarried men than women," (p.6). Besides MHAS, there is no other national data on household assets in Mexico (Wong et al., 2007). Therefore, MHAS is externally validated against estimates from Noyola (2000) the only study that provides household estimates of wealth for Mexico (Wong & Espinoza, 2002).

C. Handling of non-response and missing data

Non-response rates on wealth data are particularly high (Riphahn & Serfling, 2005) and much methodological attention has been focused on ways to reduce non-response and impute missing economic data collected from surveys. HRS has been at the forefront of these methods and the methods used by MHAS follow those pioneered by HRS (Hurd et al., 2016; Wong & Espinoza, 2004).

HRS and MHAS reduce uncertainty due to non-response by employing the unfolding brackets method. For respondents who initially do not know or refuse to provide asset values, this widely used and validated method provides a sequence of narrowing ranges (Curtin et al., 1989; Heeringa & Suzman, 1995; Juster et al., 1999). The unfolding brackets method uses the following sequence of questions. First, the respondent is asked if they own a specific asset (ownership) – this is a yes/no question. Second, the respondent is asked the value of the asset (amount). In cases where the respondent indicates holding an asset but does not provide the value of that asset they are then asked questions with "unfolding brackets." These questions begin with a large range and are asked in the form of "Is it more than $X, less than $X, or about $X." As the respondent answers, subsequent questions are asked with a narrowing range. Respondents may opt out of the question sequence at any time and therefore raw data on wealth can contain the following: valid zero-values, exact asset amounts, complete bracket responses (finished bracket sequence to the narrowest

range), incomplete bracket responses (stopped answering bracket sequence before the narrowest range), claim of ownership without information on the value (answered affirmative to ownership question but non-response to both amount and bracket questions), and unknown ownership (non-response to ownership question) (Hurd et al., 2016).

RAND HRS provides the following example to illustrate this sequence of questions: "Consider holdings of stocks and mutual funds as an example. First, the interviewer asks whether the Respondent (or her/his spouse or partner) owns any shares of stock or stock mutual funds. If affirmative, the interviewer asks the value of these stock holdings. If the Respondent is unable or unwilling to provide an exact amount, the interviewer asks whether it is more than $25,000. If the answer is "more than $25,000," the interviewer asks whether it is more than $125,000, whereas if the answer is "less than $25,000," the interviewer asks whether it is more than $2,500. Depending on the responses, the ranges are narrowed down to $0-2,500; $2,500-25,000; $25,000-125,000; $125,000-400,000; $400,000 or more," (Bugliari et al., 2019, p. 16).

After data collection, any instances where a continuous asset amount is not reported, RAND imputes the value. The unfolding brackets approach has been shown to increase accuracy of economic measurement, reduce issues with nonrandom missingness, and therefore significantly reduce the amount of imputation uncertainty (Juster & Smith, 1997). The RAND imputation process proceeds through progressive imputation steps depending on how much information is known about a particular asset. The three imputation steps correspond with the three types of missing values: (1) impute ownership if nothing is known; (2) imputation of a range, given that ownership is known or only an incomplete range is known; (3) imputation of the exact amount, given a known range. The imputation process follows in a sequence depending on level of missing values: first imputing ownership if nothing is known (1), then given ownership, imputing brackets (2), and then, given brackets, imputing exact amount (3). Recently, RAND has further reduced the variance of asset changes caused by imputation uncertainty by adding cross-wave information (previous and next wave) about asset values to the set of covariates in the imputation model (Hurd et al., 2016). Bugliari et al. (2019) details the imputation procedure and covariates for each level of missing value. MHAS uses the same process in imputing wealth (Wong et al., 2016; Wong & Espinoza, 2004). As a result of the highly regarded method (Hurd et al., 2016) used to

impute fully or partially missing data on wealth, imputed HRS and MHAS wealth data has been deemed reliable.

D. Crossnational comparability

RAND HRS and the Harmonized MHAS datasets provide harmonized wealth measures following guidelines on best practices in using wealth measures across HRS sister studies (Angrisani & Lee, 2011). Of note, they implement the following best practices: (1) collecting data on broad categories of wealth and debt and (2) collecting data at the household level. The following broad categories of wealth are collected in both studies following the example presented above in Section C: Net value of primary residence real estate, net value of vehicles, net value of businesses, net value of non-primary residence real estate, net value of financial wealth (stocks, mutual funds, investment trusts, checking accounts, savings accounts, money market accounts, CDs, bonds, t-bills), net value of other assets. Pensions are not included in the total net wealth measure because pensions and pension rights come in complex forms that differ across country contexts.

Even with the implementation of best practices in crossnational measurement of wealth and the harmonization efforts by RAND and the Gateway to Global Aging at USC, crossnational comparisons of wealth are complicated by differences in financial tools across and, differences in housing market volatility, across countries. Therefore, as a robustness check for these results I estimate the final model presented in Table 2 on non-housing financial wealth. The results are presented in Appendix 6, column 7, and show consistent results, if not evidence of greater stayer wealth disadvantage, in terms of comparative wealth between the three groups: stayers, younger returnees, and older returnees.

Overall, perfect crossnational comparability is largely unachievable. That said, comparability is not a binary state but instead a continuum. In this case the multifaceted approach to comparability including HRS and MHAS survey instruments designed for comparability, post-survey harmonization of raw data by RAND and the Gateway to Global Aging at USC, and consistent handling of incomplete data, coupled with the documented robustness check indicates that these two surveys are at the high end of crossnational comparability.

Section 3. Detailed description of data and variables

Data sources

Data comes from the RAND[2] HRS Longitudinal File 2016 (Version 1), the HRS Tracker 2016 (Early, Version 3.0) file, the Harmonized HRS (Version B,) the Rand HRS Family Data (Version 1) file, MHAS core data files, and the Harmonized MHAS (Version A). Following other studies combining HRS and MHAS (Díaz-Venegas et al., 2016; Gerst et al., 2011; Monteverde et al., 2010), for temporally comparable samples across datasets I combine MHAS data from 2001 with the HRS 2000 follow-up survey.

Asset measures

Net total assets and components. Total net assets and net asset components (taking into account debt) include: (1) net value of primary home, (2) net value of business assets, (3) net value of all other real estate assets (not including primary home), (4) net value of financial assets (including checking, savings, stocks, bonds, CDs, government savings bonds and treasury bills), (5) net value of vehicle assets.

Positive assets. Binary variables representing positive assets were constructed for each component asset type as well as total assets.

Relative assets. Assets relative to Mexican wealth are measured using a set of dummy variables representing: (1) indebtedness, (2) zero assets, (3) less than Mexican median assets, and (4) more than Mexican median assets. Assets relative to US wealth are measured using a set of dummy variables representing: (1) indebtedness, (2) zero assets, (3) less than US median assets, and (4) more than US median assets. The country medians were estimated using the total net assets of the entire sample of individuals over 50 from the HRS or MHAS, respectively. In comparison to Mexican median assets, pesos are converted to dollars using PPP to evaluate how the assets of both returnees and stayers could be used in the Mexican economy relative to all Mexican residents 50 and over. In comparison to US median assets, pesos are converted to dollars using the exchange rate to evaluate how assets could be used in the US economy relative to all US residents 50 and over.

Demographic variables

Age. Age is measured with five 5-year age brackets: 50-54, 55-59, 60-64, 65-69, 70+.

[2] The RAND HRS Longitudinal File was developed at RAND with funding from the National Institute on Aging and the Social Security Administration using HRS core data.

Gender. Gender is an important covariate because processes of migration, aging and financial well-being all differ substantively by gender. Gender is measured by a binary[3] variable indicating male or female.

Pre-migration measures

Pre-migration health. Self-reported childhood health is included to measure pre-migration health. While age at migration varies and thus childhood health may be many years before an individual immigrates, no health measurement is taken directly before immigration in either survey. The original variable is measured on a scale including excellent, very good, above average, fair, and poor. Here it is operationalized as a dichotomous measure of excellent/very good/above average health compared to fair/poor health.

Adult socioeconomic characteristics

Education. Education is correlated with labor market engagement and thus is central to socioeconomic outcomes. Education measured as a categorical variable with the following four mutually exclusive categories: (1) elementary school or less; (2) less than high school; (3) high school degree, vocational degree, or some college; (4) college or more. This variable was created by the Gateway for Global Aging at USC in order to harmonize education levels across countries. It is based on the International Standard Classification of Education 1997 (ISCED) and is valid across country contexts.

Migration

Total years in the US. Total years in the US (cumulative, including all trips) is either directly reported or constructed by subtracting the year of first migration from survey year (for stayers) or from year of last return (for returnees). In addition to measuring integration, time in the US also serves a proxy for portion of wealth accumulated in the US as this is not directly measured. Including years in the US importantly considers the portion of an individual's life course spent as an immigrant especially with the understanding that significant numbers of immigrants return within five to ten years (Constant & Massey, 2003; Dustmann & Görlach, 2016).

Age at first migration. Age at first migration is a continuous variable that is constructed from report of year of first migration to the US.

---

[3] Recognizing that gender exists on a non-binary spectrum, this analysis is limited by the common, albeit problematic, practice of measuring gender as binary.

Migrated as a child. This binary variable for migrating to the US for the first time before age 19 is constructed from the continuous measure of age at first migration. It controls for differential integration, education, labor market outcomes, and selection mechanisms of individuals who migrate as children and who are often considered 1.5 generation immigrants instead of first-generation immigrants (Portes & Rumbaut, 2001).

Years since return to Mexico. This is a continuous measure constructed from reported year of least return subtracted from year of survey. It is measured only for returnees (MHAS sample) and reported in the descriptive table. A categorical variable (stayers, returned within the last 10 years, returned more than 10 years ago) is created from this continuous measure and used in the analysis of variation in total net wealth by time since return.

Urban residence in the US. A dichotomous variable is used to represent residence in urban versus rural locations for the majority of time in the US. Those not residing in urban settings in the US are likely to have worked in agriculture a sector characterized by seasonal employment and a with higher likelihood of return.

Urban residence in Mexico. A dichotomous variable is used to represent residence in urban versus rural locations upon return to Mexico for returnees.

Period of first migration. A set of dummy variables representing first migration to the US during various policy regimes are constructed: before 1965 (Bracero era and earlier), 1965-1985, 1986 and after (following IRCA). These periods have previously been found to be differentially associated with return migration and health (Garip, 2017; Massey et al., 2002; Mueller & Bartlett, 2019).

Section 4

Table A1. Unweighted descriptive statistics and percent missing

	min	max	mean	sd	% miss.
Return status/age					---
Stayer	0	1	0.23	0.42	---
Younger returnee (< 50)	0	1	0.65	0.48	---
Older returnee (50+)	0	1	0.12	0.33	---
Age	50	99	64.74	9.49	---
50-54	0	1	0.164	0.37	---
55-59	0	1	0.184	0.388	---
60-64	0	1	0.19	0.393	---
65-69	0	1	0.176	0.381	---
70+	0	1	0.286	0.452	---
Female	0	1	0.26	0.44	---
Fair/poor childhood health	0	1	0.18	0.38	1.29%
Education	0	17	4.50	4.22	---
Elementary school or less	0	1	0.624	0.484	---
Less than high school	0	1	0.28	0.449	---
High school, vocational degree, some college	0	1	0.0445	0.206	---
College or more	0	1	0.051	0.22	---
Married	0	1	0.752	0.432	---
Total years in the US	1	87	12.68	17.24	---
Age at migration	0	49	26.28	10.07	---
Migrated as child (before age 19)	0	1	0.201	0.401	---
Years since return (returnees only)	0	81	30.06	15.97	---
Returned within last 10 years	0	1	0.14	0.35	---
Returned 10+ years ago	0	1	0.86	0.35	---
Urban residence in the US	0	1	0.55	0.50	0.57%
Urban residence in Mexico at return (returnees only)	0	1	0.67	0.01	---
Period of first migration					---
Before 1965	0	1	0.58	0.02	---
1965-1985	0	1	0.35	0.01	---
1986+	0	1	0.07	0.01	---
Total net wealth (PPP)	-19,354	871,317	33,899	60,589	---
Total net wealth (exchange rate)	-13,815	621,974	26,467	47,947	---
Distribution of debt, zero, positive assets					---
Debt	0	1	0.0136	0.116	---
Zero assets	0	1	0.0474	0.213	---
Positive net assets	0	1	0.939	0.24	---

Sources: HRS 2000, MHAS 2001

Section 5. Inverse hyperbolic sine (IHS) transformation

The inverse hyperbolic sine (IHS) transformation is often used to transform dependent variables with right-skewed distributions, like wealth. The advantage of an IHS transformation over a log transformation is that IHS does not require discarding non-positive values, which are substantively meaningful in analyses of wealth and income zero. However, IHS introduces two challenges. First, IHS is not invariant to scaling (Aihounton & Henningsen, 2021). This means that changing the unit of measurement of the IHS transformed outcome variable can alter regression coefficients (on the IHS scale) and marginal effects (on the original scale). Second, IHS transformed regression coefficients need to be retransformed to the original scale in order to report and analyze meaningful marginal effects (Norton, 2022).

To address the first challenge – scaling – I follow the recommendations of Aihounton and Henningsen (2021). Aihounton and Henningsen use a Monte-Carlo simulation to compare 14 ways to assess alternate scales, across three types of criteria: (1) criteria assessing the 'fit' of the regression model, (2) criteria assessing the distribution of the regression residuals, and (3) criteria assessing appropriateness of model assumptions residuals. They conclude that with a IHS transformed dependent variable, the R-squ predictive R-squ criteria perform best across all scenarios, and that the skewness of residuals, Breusch–Pagan test, and Ramsey's Regression Equation Specification Error Test (RESET) test perform "reasonably well" (p. 344) and recommend their use as additional criteria. Following this procedure, I calculate all six models adjusting the dependent variable (PPP converted, IHS transformed net wealth) by scales ranging from one-millionth (0.000001) to 1 million (1,000,000) and compare the model R-squ, skewness of residuals, Ramsey's Regression Equation Specification Error Test (RESET) test, and the Breusch–Pagan test. These criteria include at least one from each of the three types of criteria (fit, distribution of residuals, model assumptions). I find that scaling PPP converted, IHS transformed net wealth by .001 is the optimal scaling across most of the six models and four criteria and is thus used in this analysis. Other scales served to increase the wealth advantage of both groups of returnees and therefore the results presented here, with the .001 scaling factor, are potentially a conservative estimate of differences in wealth by return group.

The second challenge of using an IHS transformation is that coefficients are not directly interpretable and are not semi-elasticities like they are when using a log transformation. Therefore, for accurate retransformation out of IHS to calculate marginal effects, Norton (2022) recommends using Duan's (1983) smearing estimator. Duan's smearing estimator does not assume a normal distribution

of log-scale errors. Therefore, after estimating each model using the scaled (.001) and IHS transformed dependent variable (PPP converted, IHS transformed net wealth), I use Duan's smearing to retransform out of IHS and back to the original scale (2000 dollars). The marginal effects in the original scale, after undertaking these procedures, are presented in Figure 4.

Section 6

Table A2. Sensitivity checks for robustness to analytic decisions, fully adjusted model (OLS predicting IHS transformed net wealth)

	(1) Final model	(2) Sample > 18	(3) In US 2+ yrs	(4) In US 3+ yrs	(5) Return at age 55+	(6) Return at age 60+	(7) Financial assets
Return group (ref: stayers)							
Return before age 50	0.61*	0.69*	0.84**	0.79**	0.55*	0.59*	1.55***
	(0.27)	(0.33)	(0.29)	(0.29)	(0.27)	(0.27)	(0.24)
Return at 50 or older	0.65*	0.81*	0.89**	0.83**	0.83**	0.78*	1.50***
	(0.27)	(0.32)	(0.28)	(0.28)	(0.29)	(0.32)	(0.22)
Migrated before age 19	0.34*		0.26	0.23	0.36*	0.35*	0.18
	(0.16)		(0.18)	(0.20)	(0.16)	(0.16)	(0.18)
Urban residence US	0.04	-0.02	0.01	0.00	0.04	0.03	0.47***
	(0.14)	(0.17)	(0.17)	(0.18)	(0.14)	(0.14)	(0.12)
Missing urban residence US	0.25	0.90**	-0.12	-0.22	0.30	0.28	-0.59
	(0.54)	(0.31)	(0.88)	(0.98)	(0.53)	(0.53)	(0.31)
Age (ref: 50-54)							
55-59	-0.13	-0.18	-0.25	-0.38	-0.14	-0.13	-0.05
	(0.21)	(0.24)	(0.24)	(0.26)	(0.21)	(0.21)	(0.23)
60-64	-0.25	-0.37	-0.43	-0.59*	-0.26	-0.25	-0.02
	(0.20)	(0.24)	(0.23)	(0.24)	(0.20)	(0.20)	(0.20)
65-69	-0.33	-0.44	-0.70**	-0.86**	-0.34	-0.33	0.03
	(0.21)	(0.25)	(0.25)	(0.27)	(0.21)	(0.22)	(0.20)
70+	-0.32	-0.35	-0.66**	-0.70**	-0.33	-0.33	0.04
	(0.20)	(0.24)	(0.24)	(0.23)	(0.20)	(0.20)	(0.19)
Female	-0.09	-0.16	0.04	-0.07	-0.09	-0.09	0.22
	(0.18)	(0.21)	(0.19)	(0.19)	(0.18)	(0.18)	(0.14)
Married	0.68***	0.62**	0.91***	0.82***	0.67***	0.67***	0.05
	(0.18)	(0.21)	(0.22)	(0.22)	(0.18)	(0.18)	(0.15)
Fair/poor health as child	-0.05	-0.06	-0.07	-0.06	-0.04	-0.04	-0.17
	(0.19)	(0.21)	(0.26)	(0.29)	(0.19)	(0.19)	(0.11)
Missing health as child	0.45	0.35	0.80*	0.89*	0.47	0.46	-0.56
	(0.36)	(0.38)	(0.40)	(0.44)	(0.38)	(0.37)	(0.34)
Education (ref: elementary or less)							

Less than high school	0.30	0.25	0.23	0.23	0.30*	0.30	0.32*
	(0.15)	(0.18)	(0.18)	(0.19)	(0.15)	(0.15)	(0.14)
High school, vocational or some college	0.89**	0.63	0.80**	0.75*	0.89**	0.89**	1.04***
	(0.27)	(0.44)	(0.29)	(0.30)	(0.27)	(0.27)	(0.28)
College or more	1.89***	2.02***	1.54*	1.66**	1.89***	1.89***	2.23***
	(0.47)	(0.58)	(0.70)	(0.62)	(0.47)	(0.47)	(0.33)
Total years in US	0.01	0.01	0.02*	0.02*	0.01	0.01	0.01*
	(0.01)	(0.01)	(0.01)	(0.01)	(0.01)	(0.01)	(0.01)
Constant	2.19***	2.28***	2.01***	2.28***	2.25***	2.22***	-1.12**
	(0.38)	(0.45)	(0.45)	(0.44)	(0.38)	(0.39)	(0.36)

standard errors in parentheses

*** p<0.001, ** p<0.01, * p<0.05

Section 7

Table A3. OLS regression coefficients predicting IHS transformed total net wealth - ***including retirement accounts in HRS*** (weighted)

	(1) Unadjusted	(2) Demographic	(3) Pre-migration	(4) Socioeconomic	(5) Migration	(6) Fully adjusted
Return group (ref: stayers)						
Return before age 50	0.21	0.34	0.35	0.23	0.58	0.57*
	(0.16)	(0.17)	(0.18)	(0.14)	(0.32)	(0.28)
Return at 50 or older	0.35	0.51*	0.51*	0.43*	0.66*	0.62*
	(0.22)	(0.20)	(0.20)	(0.21)	(0.27)	(0.27)
Migrated before age 19		0.60***	0.59***	0.45**	0.51**	0.32*
		(0.15)	(0.15)	(0.15)	(0.17)	(0.16)
Urban residence US		0.30	0.31	0.05	0.27	0.01
		(0.17)	(0.17)	(0.15)	(0.18)	(0.15)
Missing urban residence US		0.96***	0.96***	0.35	0.89***	0.24
		(0.21)	(0.21)	(0.48)	(0.24)	(0.55)
Age (ref: 50-54)						
55-59		-0.10	-0.10	-0.13	-0.09	-0.11
		(0.23)	(0.23)	(0.21)	(0.23)	(0.21)
60-64		-0.22	-0.22	-0.22	-0.24	-0.24
		(0.21)	(0.21)	(0.20)	(0.21)	(0.20)
65-69		-0.28	-0.28	-0.29	-0.31	-0.33
		(0.24)	(0.24)	(0.21)	(0.25)	(0.21)
70+		-0.28	-0.28	-0.24	-0.34	-0.32
		(0.19)	(0.19)	(0.19)	(0.21)	(0.20)
Female		-0.06	-0.06	-0.10	-0.05	-0.07
		(0.20)	(0.20)	(0.18)	(0.20)	(0.18)
Married		0.69***	0.69***	0.69***	0.70***	0.69***
		(0.20)	(0.20)	(0.18)	(0.20)	(0.19)
Fair/poor health as child			-0.09			-0.06
			(0.20)			(0.19)
Missing health as child			0.43			0.46
			(0.35)			(0.37)
Education (ref: elementary or less)						

Less than high school				0.32*		0.31*
				(0.16)		(0.16)
High school, vocational or some college				1.00***		1.02***
				(0.30)		(0.30)
College or more				1.90***		1.92***
				(0.47)		(0.48)
Total years in US					0.01	0.01
					(0.01)	(0.01)
Constant	3.24***	2.52***	2.52***	2.53***	2.30***	2.23***
	(0.13)	(0.34)	(0.34)	(0.31)	(0.43)	(0.39)

standard errors in parentheses

*** $p<0.001$, ** $p<0.01$, * $p<0.05$

Section 8. Supplementary References

Aihounton, G. B. D., & Henningsen, A. (2021). Units of measurement and the inverse hyperbolic sine transformation. *The Econometrics Journal*, *24*(2), 334–351.

Angrisani, M., & Lee, J. (2011). *Harmonization of cross-national studies of aging to the health and retirement study. Wealth measures* (Working Paper No. WR-861/6). Santa Monica, CA: Rand Corporation

Bosworth, B., & Smart, R. (2009). Evaluating micro-survey estimates of wealth and saving. (CRR Working Paper, No. 2009-4). Chestnut Hill, MA: Center for Retirement Research at Boston College. http://dx.doi.org/10.2139/ssrn.1368830

Bugliari, D., Campbell, N., Chan, C., Hayden, O., Hayes, J., Hurd, M., Karabatakis, A., Main, R., Mallett, J., & McCullough, C. (2019). RAND HRS detailed imputations file 2016 (V1) documentation. Santa Monica, CA: *RAND Center for the Study of Aging.*

Constant, A. F., & Massey, D. S. (2003). Self-selection, earnings, and out-migration: A longitudinal study of immigrants to Germany. *Journal of Population Economics*, *16*(4), 631–653.

Curtin, R. T., Juster, T., & Morgan, J. N. (1989). Survey estimates of wealth: An assessment of quality. In R. E. Lipsey & H. Stone Tice (Eds.), NBER *Studies in Income and Wealth: Vol. 52 The measurement of saving, investment, and wealth* (pp. 473–552). Chicago, IL: University of Chicago Press.

Díaz-Venegas, C., Reistetter, T. A., & Wong, R. (2016). Differences in the Progression of Disability: A U.S.–Mexico Comparison. *The Journals of Gerontology Series B: Psychological Sciences and Social Sciences*, 73: 913-922.

Duan, N. (1983). Smearing estimate: A nonparametric retransformation method. *Journal of the American Statistical Association*, *78*(383), 605–610.

Dustmann, C., & Görlach, J.-S. (2016). The economics of temporary migrations. *Journal of Economic Literature*, *54*(1), 98–136.

Fries, G., Starr-McCluer, M., Sunden, A. E., Maki, D., Montalto, C., & Moore, K. (1998, March). The measurement of household wealth using survey data: An overview of the Survey of Consumer

Finances. Paper presented at the annual conference of the American Council of Consumer Interests, Washington, DC.

Garip, F. (2017). *On the move: Changing mechanisms of Mexico-U.S. migration* (Vol. 2). Princeton, NJ: Princeton University Press.

Gerst, K., Michaels-Obregon, A., & Wong, R. (2011). The impact of physical activity on disability incidence among older adults in Mexico and the United States. *Journal of Aging Research*, *2011,* 420714.

Gibson-Davis, C. M., & Percheski, C. (2018). Children and the elderly: Wealth inequality among America's dependents. *Demography*, *55*, 1009–1032.

Heeringa, S. G., & Suzman, R. (1995). *Unfolding brackets for reducing item nonresponse in economic surveys*. (Health and Retirement Study working paper). Ann Arbor, MI: Survey Research Center, Institute for Social Research, University of Michigan.

Hurd, M. D., Meijer, E., Moldoff, M., & Rohwedder, S. (2016). *Improved wealth measures in the Health and Retirement Study: Asset reconciliation and cross-wave imputation* (Report). Santa Monica, CA: Rand Corporation.

Juster, F. T., & Smith, J. P. (1997). Improving the Quality of Economic Data: Lessons from the HRS and AHEAD. *Journal of the American Statistical Association*, *92*(440), 1268–1278.

Juster, F. T., Smith, J. P., & Stafford, F. (1999). The measurement and structure of household wealth. *Labour Economics*, *6*(2), 253–275.

Keister, L. A. (2011). *Faith and money: How religion contributes to wealth and poverty*. New York, NY: Cambridge University Press.

Keister, L. A. (2014). The one percent. *Annual Review of Sociology*, *40*, 347–367.

Kennickell, A. B. (2008). The Role of Over-sampling of the Wealthy in the Survey of Consumer Finances. *Irving Fisher Committee Bulletin*, *28*(August), 403–408.

Litwak, E., & Longino C. F., Jr. (1987). Migration patterns among the elderly: A developmental perspective. *The Gerontologist*, *27*(3), 266–272.

Massey, D. S., Durand, J., & Malone, N. J. (2002). *Beyond smoke and mirrors: Mexican immigration in an era of*

economic integration. New York, NY: Russell Sage Foundation.

Michaels-Obregon, A., Wong, R., Phillips, D., Wilkens, J., & Lee, J. (2022). *Harmonized MHAS Documentation.* (Report). Los Angeles, CA: USC Dornsife Center for Economic and Social Research, Program on Global Aging, Health, and Policy.

Monteverde, M., Noronha, K., Palloni, A., & Novak, B. (2010). Obesity and excess mortality among the elderly in the United States and Mexico. *Demography*, *47*(1), 79–96.

Mueller, C. W., & Bartlett, B. J. (2019). U.S. Immigration Policy Regimes and Physical Disability Trajectories Among Mexico–U.S. Immigrants. *The Journals of Gerontology, Series B*: *Psychological Sciences and Social Sciences 74*, 725–734.

Norton, E. C. (2022). *The Inverse Hyperbolic Sine Transformation and Retransformed Marginal Effects*. (NBER Working Paper 29998). Cambridge, MA: National Bureau of Economic Research. Retrieved from https://www.nber.org/system/files/working_papers/w29998/w29998.pdf

Noyola, A. J. (2000). *The distribution of household wealth in Monterrey, Mexico in the 1990's* (Unpublished doctoral dissertation). University of Notre Dame, Notre Dame, IN.

Portes, A., & Rumbaut, R. G. (2001). *Legacies: The story of the immigrant second generation*. Berkeley, CA: University of California Press.

Riphahn, R. T., & Serfling, O. (2005). Item non-response on income and wealth questions. *Empirical Economics*, *30*(2), 521–538.

Sierminska, E., Michaud, P.-C., & Rohwedder, S. (2008, November). *Measuring wealth holdings of older households in the US: A comparison using the HRS, PSID and SCF*. Paper presented at the Pensions, Private Accounts, and Retirement Savings Over the Life Course workshop, Ann Arbor, MI.

Wolff, E. N. (2016). Household wealth trends in the United States, 1962 to 2013: What happened over the Great Recession? *RSF: The Russell Sage Foundation Journal of the Social Sciences*, *2*(6), 24–43.

Wong, R., & DeGraff, D. S. (2009). Old-age wealth in Mexico: The role of reproductive, human capital, and employment decisions. *Research on Aging*, *31*(4), 413–439.

Wong, R., & Espinoza, M. (2002, November). Economic status of middle and old age population in

Mexico: Preliminary results from the Mexican Health and Aging Study. Paper presented at the Gerontological Society of America conference, Boston, MA.

Wong, R., & Espinoza, M. (2004). Imputation of non-response on economic variables in the Mexican Health and Aging Study (MHAS/ENASEM) 2001 (Project Report). Retrieved from https://mhasweb.org/Resources/DOCUMENTS/2001/Imputation_of_Non-Reponse_on_Economic_Variables_in_the_MHAS-ENASEM_2001.pdf.

Wong, R., Orozco-Rocha, K., Zhang, D., Michaels-Obregon, A., & Gonzalez-Gonzalez, C. (2016). *Imputation of Non-Response on Economic Variables in the Mexican Health and Aging Study (MHAS/ENASEM) 2012* (Project Report). Retrieved from https://mhasweb.org/Resources/DOCUMENTS/2012/Imputations/Imputation_of_Non_Response_on_Economic_Variables_in_the_MHAS_ENASEM_2012.pdf

Wong, R., Palloni, A., & Soldo, B. J. (2007). Wealth in middle and old age in Mexico: The role of international migration. *International Migration Review*, *41*(1), 127–151.
